# Understanding Multi‐Scale and Multi‐Species Habitat Selection by Mammals in the Eastern Himalayan Biodiversity Hotspot

**DOI:** 10.1002/ece3.71247

**Published:** 2025-04-23

**Authors:** Arif Ahmad, Govindan Veeraswami Gopi

**Affiliations:** ^1^ Wildlife Institute of India Dehradun India

**Keywords:** Arunachal Pradesh, biodiversity hotspot, biomod2, FRAGSTAT, habitat fragmentation, landscape, protected areas, species distribution modeling

## Abstract

Human‐induced habitat loss and fragmentation threaten biodiversity in the Eastern Himalayas, a crucial part of the Indo‐Myanmar biodiversity hotspot. This study examines the distribution of 10 mammal species in Arunachal Pradesh using a multi‐scale ensemble modeling approach, integrating Generalized Linear Models (GLM), Generalized Additive Models (GAM), and MaxEnt to assess habitat suitability. By analyzing 57 environmental predictor variables across multiple spatial scales, we found that elevation is a key determinant for carnivores such as the dhole and the Asiatic golden cat, while herbivores like the northern red muntjac and the mainland serow prefer broadleaf forests. Species distributions showed distinct patterns, with most carnivores concentrated in the south, except for the widely distributed yellow‐throated marten. Dhole and leopard cat preferred elevated broadleaf forests, while the Asiatic golden cat favored mixed forests. Herbivores like the northern red muntjac and mainland serow were found at higher elevations, whereas the Indian wild pig preferred grasslands and degraded habitats near human settlements. While protected areas (PAs) exhibited higher species richness, significant suitable habitats also exist outside these regions, underscoring the need for landscape‐level conservation strategies. Precipitation seasonality and human population density emerged as significant predictors, highlighting the influence of climatic and anthropogenic factors on habitat suitability. Our findings emphasize the necessity of conserving large, connected landscapes to mitigate human‐induced pressures and climate change impacts on these species. By combining spatial modeling with ecological insights, this study provides a framework for prioritizing conservation efforts. Future research should expand data collection across broader temporal and geographic scales and incorporate climate change projections to anticipate species distribution shifts. These findings are critical for guiding effective conservation planning and habitat management in this ecologically rich yet vulnerable region.

## Graphical abstract

This study assesses habitat suitability for 10 mammal species in Arunachal Pradesh, a biodiversity hotspot in the Eastern Himalayas, using a multi‐scale modeling approach to examine the impacts of human‐induced landscape changes on species distribution, focusing on rare and threatened mammals. This research provides critical insights for conservation efforts in the region.

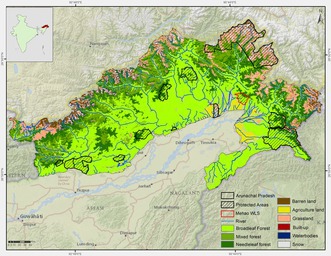

## 1 Introduction

Human‐induced habitat loss and degradation pose a significant threat to biodiversity (Newbold et al. 2014). As the global population grows, increasing land‐use changes and habitat fragmentation further intensify these challenges (Hurtt et al. 2011; Krausmann et al. 2013). Understanding the impacts of habitat fragmentation on wildlife communities is essential for developing effective conservation strategies (Gonzalez et al. 1998; Crooks 2002). Over 50% of the world's land cover is affected by rising human activity, population growth, and increased per capita resource use (McGill et al. 2015). Human‐induced changes, including land use and climate change, have transformed nearly every ecosystem on Earth (Hansen et al. 2001). The rapid pace of these changes presents a challenge for wildlife species adapting to altered landscapes (Myers and Knoll 2001). Activities such as agriculture, urban expansion, and transportation infrastructure adversely affect habitat quality and availability (Dixo et al. 2009), impacting species' survival (Cushman et al. 2018; Kaszta et al. 2019), dispersal (Cushman et al. 2014; Mateo‐Sanchez et al. 2014), and gene flow (Scolozzi and Geneletti 2012; Gao et al. 2013).

The survival of species populations in human‐altered environments significantly affects biodiversity (Gardner et al. 2009). Biotic and abiotic factors such as food availability, predation, guild interactions, and competition influence how animals use and navigate their environments (Falkenberg and Clarke 1998). To effectively conserve and manage habitats, it is crucial to understand the factors influencing space utilization of the species. Species distribution modeling (SDM) is a foundation for quantifying species‐environment relationships, predicting species' spatial distributions, and conservation planning (Macdonald et al. 2018, 2019; Miller 2010). These models help to identify suitable habitats and assess how species may respond to environmental changes such as climate shifts or habitat degradation (Guisan et al. 2013). In conservation science, SDMs are particularly valuable for guiding efforts to protect biodiversity in landscapes and protected areas (Phillips et al. 2006). Ensemble modeling, an approach within SDM, involves combining predictions from multiple models to increase the robustness and accuracy of species distribution predictions (Araújo and New 2007). By integrating results from various algorithms, ensemble models minimize the biases and uncertainties inherent in individual modeling techniques (Thuiller et al. 2009). This approach proves essential in addressing the complexities of species‐environment interactions across varied landscapes, ensuring more reliable conservation planning (Marmion et al. 2009).

Arunachal Pradesh, located in the Eastern Himalayas and recognized as a biodiversity hotspot, supports a rich assemblage of species with diverse ecological needs. However, many of these species face increasing threats from habitat fragmentation, human activities, and climate change (Bharali and Khan 2011). The region is home to 105 mammal species and subspecies, spanning 85 genera, 25 families, and 9 orders (Editor‐Director 2006a, 2006b). Despite this remarkable diversity, comprehensive information on mammal distribution remains limited. Existing research has primarily focused on the status and distribution of primates (Sarma et al. 2015, 2021; Sarania et al. 2017), leaving significant gaps in understanding the habitat preferences and spatial distribution of other mammalian species. To bridge this gap, we applied a multi‐scale ensemble modeling approach to evaluate the suitability of 10 key mammal species across different spatial scales. The selection of species for this study was based on data availability, their ecological significance, and their relevance to conservation efforts in the region. These 10 species include the northern red muntjac (*Muntiacus vaginalis*), Asiatic golden cat ( *Catopuma temminckii* ), leopard cat ( *Prionailurus bengalensis* ), mainland serow ( *Capricornis thar* ), common palm civet ( *Paradoxurus hermaphroditus* ), masked palm civet ( *Paguma larvata* ), large Indian civet ( *Viverra zibetha* ), dhole ( *Cuon alpinus* ), Indian wild pig ( *Sus scrofa* ), and yellow‐throated marten ( *Martes flavigula* ). Notably, the IUCN Red List classifies the dhole as Endangered, the Asiatic golden cat as Near Threatened, and the mainland serow as Vulnerable.

With growing global attention on biodiversity conservation, especially in hotspots like Arunachal Pradesh, using an ensemble modeling approach in SDM is vital for developing effective conservation plans (Malakoutikhah et al. 2020). These tools not only help protect species but also ensure that biodiversity conservation efforts remain resilient to the challenges posed by climate change and land‐use alterations (Rathore et al. 2022). This study considered both broad‐scale and fine‐scale habitat selection. Recognizing the scale‐dependent nature of species responses to environmental factors, we highlight the importance of including appropriate variables for specific model scales (McGarigal et al. 2016; Wan et al. 2017; Klaassen and Broekhuis 2018; Khosravi et al. 2019; Atzeni et al. 2020; Stuber and Gruber 2020; Rather et al. 2020a, 2020b; Ash et al. 2021; Dar et al. 2021, Cushman et al. 2024).

This study has three objectives: (1) to assess the impact of environmental variables and landscape patterns on species distribution across different spatial scales; (2) to predict species distribution in previously unexamined areas using an ensemble modeling approach and compare suitable habitat areas inside and outside Protected Areas (PAs); and (3) to determine the conservation needs of multiple species across these scales. By achieving these objectives, this study aims to provide valuable insights into the habitat requirements and conservation priorities for rare and elusive mammalian species in Arunachal Pradesh. These findings will support the development of targeted landscape‐scale management strategies that promote the persistence and recovery of these species. This work will also contribute to more effective conservation planning in this ecologically rich and diverse region.

## 2 Study Area

**FIGURE 1:**
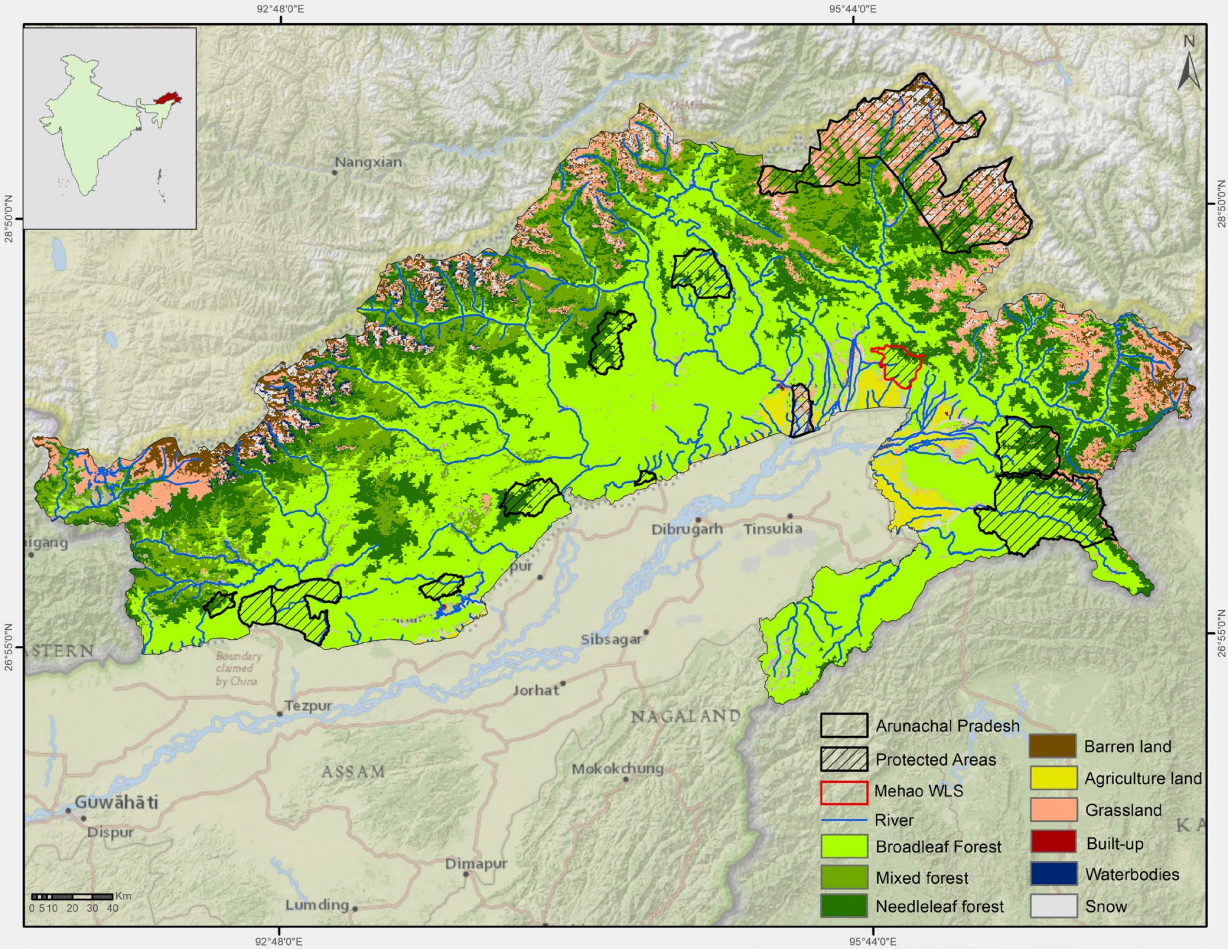
Location map of Arunachal Pradesh with the land cover classes and protected areas.

Arunachal Pradesh, situated between 26°28′ and 29°30′ N latitude and 91°30′–97°30′ E longitude, is an Eastern Himalayan state (Figure 1) and encompasses a total area of 83,700 km^2^. It shares borders with Bhutan to the west, Tibet to the north, Myanmar to the east, and the northeastern Indian states of Assam and Nagaland to the south. This region serves as an ecological bridge between the Palearctic and Indo‐Malayan biogeographic realms, supporting species adapted to both temperate and tropical environments (Mani 1974; Rodgers and Panwar 1988; Singh et al. 2007). Arunachal Pradesh is one of the 200 most significant ecoregions globally and falls within the Indo‐Myanmar biodiversity ‘Hotspots' (Myers et al. 2000; Olson and Dinerstein 1998) and encompasses 11 Wildlife Sanctuaries and 2 National Park (Wildlife Institute of India n.d.). The state plays a crucial role in maintaining habitat connectivity across the Eastern Himalayas, acting as a corridor for migratory species and ensuring gene flow among wildlife populations (Sarma et al. 2021). The state also supports an exceptional diversity of birds, amphibians, and reptiles, contributing to its status as a critical region for global biodiversity conservation (Singh et al. 2007). It also forms part of the Eastern Himalayan Endemic Bird Area (Collar et al. 1994). A major part of the state is hilly and mountainous. The altitude varies considerably, from 130 m in the lowlands to over 6000 m in the high mountains. The climate of Arunachal Pradesh is mainly tropical ‘monsoon’ type with a hot wet summer and a cool dry winter with an average temperature ranging from less than 0°C to 35°C (Choudhury 1999) and mean annual rainfall was found to be 2345 mm (Bhagawati et al. 2016).

Arunachal Pradesh features a wide range of habitats, with its unique geographical extent contributing to a rich diversity of flora and fauna. It is recognized for hosting nearly half of all flowering plant species in India and boasting the northernmost tropical rainforest worldwide (Rao and Hajra 1986; Choudhury 1999; Proctor et al. 1998; Whitmore et al. 1998). This region encompasses diverse ecosystems, ranging from tropical rainforests and subtropical broadleaf forests to temperate forests and alpine meadows, each supporting unique assembles of species adapted to varying climatic conditions and altitudinal gradients (Choudhury 1999; Proctor et al. 1998). The state is estimated to host over 5000 species of flowering plants, including vascular and non‐vascular varieties (Bhatnagar et al. 2017). This rich floral diversity includes 238 endemic species, 600 species of orchids, 89 species of bamboo, 18 species of canes, 400 species of ferns, 24 species of gymnosperms, as well as a high diversity of fungi, lichens, and bryophytes.

The state is home to 26 indigenous communities across 28 districts, each with distinct cultural traditions and deep‐rooted connections to the land. Around 80% of these communities practice shifting agriculture and depend on wild meat for subsistence (Hilaluddin and Ghose 2005; Elwin 1959). However, rapid demographic and socio‐economic changes, including a 26% population increase between 2001 and 2011 (Census of India 2011), have altered human‐environment interactions. These changes have escalated human‐induced pressures on fragile ecosystems, leading to critical environmental issues such as habitat fragmentation, increased deforestation, and overexploitation of wildlife, posing significant threats to biodiversity conservation.

## 3 Methodology

### 3.1 Species Occurrence Records and Spatial Autocorrelation

We collected spatial occurrence data through reconnaissance, camera‐trap surveys, and prior systematic research. To ensure an accurate representation of species presence, we used georeferencing techniques to extract location information from prior systematic research. We georeferenced several datasets using ArcGIS version 10.3, as detailed in Appendix S1. This appendix lists the georeferenced data sources used in the study, primarily reports and research papers that provide valuable information on the distribution and status of various species in protected areas of Arunachal Pradesh. To document the presence of mammalian species, we conducted field surveys in and around Mehao Wildlife Sanctuary in two phases: October 2017–April 2018 and November 2019–February 2020. We recorded species presence using Garmin 20 eTrex GPS (Global Positioning System) units, based on direct sightings and indirect evidence during reconnaissance surveys. Additionally, we deployed camera trap (Cuddeback C1) units randomly across the study area, setting up 58 trap locations with one trap per 3 sq. km grid and an average spacing of 1.2–1.5 km between traps. The camera traps, equipped with a 1.4‐s trigger speed, a 100‐ft detection range, and fast‐as‐possible (FAP) programming, were placed 20 cm above the ground along animal trails. These traps operated continuously to capture images of mammals. Our main objective for the camera trap sampling was to estimate the relative detection rate and occupancy of mammals in and around Mehao Wildlife Sanctuary (Ahmad and Gopi 2024).

**FIGURE 2:**
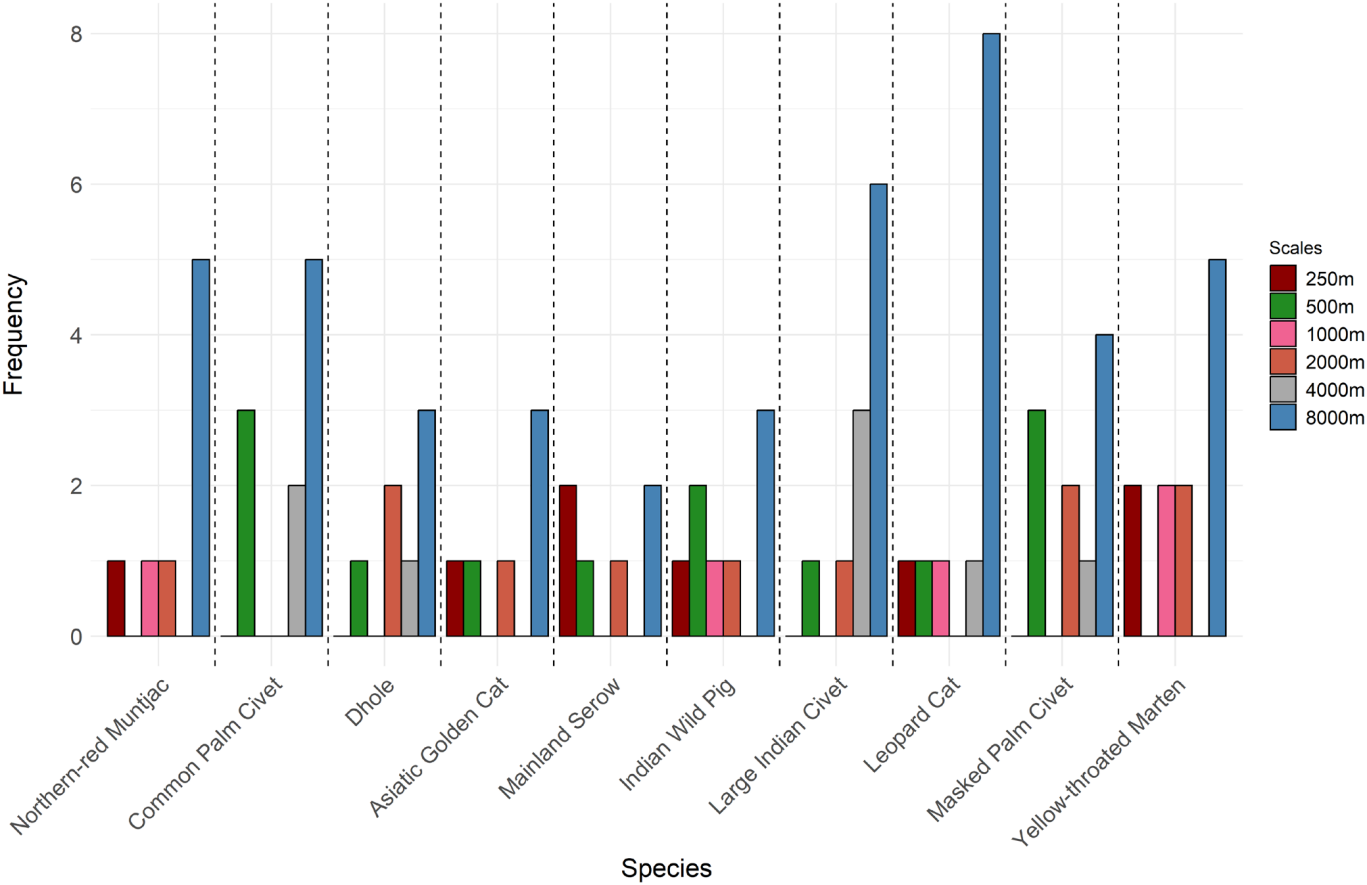
Frequency of selected spatial scales (in meters) across all variables for the ten mammalian species analyzed.

We employed two methods to address spatial autocorrelation, a known bias associated with spatial data. First, we used the SDM toolbox in ArcGIS version 10.3 (Environmental Systems Research Institute 2014) to apply spatial filtering, which reduced spatial bias in species presence data. Second, we calculated Global Moran's‐I using ArcGIS's Spatial Autocorrelation tool to assess whether the spatially rarefied occurrence data exhibited a random distribution after spatial filtering (Moran 1950). After addressing spatial bias for all 10 species (Table 1), we retained a set of spatially rarefied occurrence records for further analysis. The final dataset included 93 records for dhole (from an initial 132), 55 for Asiatic golden cat (from 73), 135 for leopard cat (from 179), 48 for Himalayan palm civet (from 57), 153 for Northern red muntjac (from 240), 147 for Mainland serow (from 194), 26 for Common palm civet (from 41), 43 for Large Indian civet (from 66), 78 for Yellow‐throated marten (from 95), and 80 for Wild pig (from 103). Figure 2 presents the geo‐coordinates of the species' spatially rarefied occurrence records.

**TABLE 1:** Ten study species and their IUCN status.

Order	Family	Species	IUCN status	Reference
Carnivora	Felidae	Asiatic Golden Cat ( *Catopuma temminckii* )	VU	McCarthy et al. (2015)
		Mainland Leopard Cat ( *Prionailurus bengalensis* )	LC	Ghimirey et al. (2023)
	Canidae	Asiatic Wild Dog ( *Cuon alpinus* )	EN	Kamler et al. (2015)
	Mustelids	Yellow‐throated marten ( *Martes flavigula* )	LC	Chutipong et al. (2024)
	Viverrids	Masked Palm Civet ( *Paguma larvata* )	LC	Duckworth, Timmins, Chutipong, et al. (2024)
		Large Indian Civet ( *Viverra zibetha* )	VU	Timmins et al. (2024)
		Common Palm Civet ( *Paradoxurus hermaphroditus* )	LC	Duckworth, Timmins, Choudhury, et al. (2024)
Artiodactyla	Bovidae	Himalayan Serow ( *Capricornis thar* )	NT	Phan et al. (2020)
	Cervidae	Northern‐red Muntjac (*Muntiacus vaginalis*)	LC	Timmins et al. (2016)
	Suidae	Indian Wild Pig ( *Sus scrofa* )	LC	Keuling and Leus (2019)

Abbreviations: EN, endangered; LC, least concern; NT, near threatened; VU, vulnerable.

### 3.2 Environmental Predictors

The relationship between species and their habitat was established by considering 57 predetermined variables, categorized into five broad groups: climate, topography, landscape metrics, vegetation, and human influence. All variables undertook resampling in ArcGIS to achieve a spatial resolution of 250 m and were projected to the 46 N UTM projection. Continuous variables were resampled using a bilinear interpolation approach, while categorical variables employed the nearest neighborhood approach. A comprehensive list of these variables, including their sources and descriptions, can be found in Table 2.

**TABLE 2:** Description of 55 predictor variables used in multi‐scale habitat modeling to predict the potential distribution of Asiatic golden cat, dhole, leopard cat, large Indian civet, masked palm civet, common palm civet, yellow‐throated marten, northern red muntjac, mainland serow, and Indian wild pig.

Predictor variable	Description	Units	Variable justification	Source
Group 1: Climate				
Actual evapotranspiration (AET)	Mean monthly estimates	mm/month	The CGIAR‐CSI database provides standardized Actual Evapotranspiration (AET) data, essential for modeling mammal habitats by indicating water availability and vegetation productivity. AET captures water balance and seasonal dynamics, influencing habitat suitability and migration patterns (Mohammed and Mohd Sah 2024; Quiroga‐Pacheco et al. 2024; Gupta and Krishnamurthy 2023)	CGIAR‐CSI database (http://www.cgiar‐csi.org)
AET of summer	Calculated using mean monthly estimates	mm/month		
AET of winter		mm/month		
Annual potential evapotranspiration (PET)	Mean monthly estimates	mm/month	These seasonal variables help represent key ecological constraints, like vegetation dynamics and water availability, essential for species distributions, especially in variable climates (Mohammed and Mohd Sah 2024; Title and Bemmels 2018). The selection of environmental variables enhances model robustness, leading to more reliable predictions of the habitat suitability model (Quiroga‐Pacheco et al. 2024; Afolayan and Ajayi 1980)	ENVIREM (http://envirem.github.io; Title and Bemmela 2017)
PET seasonality	Monthly variability in potential evapotranspiration	mm/month		
PET of coldest quarter	Mean monthly PET of coldest quarter	mm/month		
PET of driest quarter	Mean monthly PET of driest quarter	mm/month		
PET of warmest quarter	Mean monthly PET of warmest quarter	mm/month		
PET of wettest quarter	Mean monthly PET of wettest quarter	mm/month		
Maximum temperature of the coldest month	Maximum temperature of the coldest month, i.e., January	°C		
Minimum temperature of the warmest month	Minimum temperature of the warmest month, i.e., June	°C		
BioClim 1–19			The BIOCLIM dataset provides bioclimatic variables derived from temperature and precipitation data, essential for modeling species distributions. BIOCLIM captures both annual climatic trends and extremes, offering a comprehensive understanding of environmental gradients affecting species' ranges, such as thermal limits and water availability (Richmond et al. 2010; Phillips et al. 2006). Mammals depend on climatic conditions for habitat, forage, and water (Deb et al. 2020). Variables like mean annual temperature, precipitation of the driest month, and temperature seasonality directly influence vegetation and habitat quality (Khlyap et al. 2023; Morales‐Barbero and Vega‐Álvarez 2019)	WorldClim (https://www.worldclim.org/; Hijmans et al. 2005)
Snow and ice cover	Land cover type at 500 m resolution (MCD12Q1, version 051, year 2010)	%	Snow and ice coverage in the Himalayas shape the distribution and seasonal behavior of species (Kichloo et al. 2023). Snow cover directly influences vegetation growth, which in turn affects prey and predators (Singh et al. 2020; Ghoshal et al. 2019). Additionally, snow and ice patterns help define the distribution of water sources that are critical for all species, particularly during the dry winter months when snowmelt is the primary water source	MODIS (https://lpdaac.usgs.gov)
Group 2: Vegetation				
Land cover and vegetation indices				
Normalized vegetation index (NDVI)	16‐day NDVI at 1 km resolution (MOD13A2, version 005, years 2002–2016)	—	NDVI in different seasons captures seasonal variations in vegetation, which directly affects the availability of food and shelter for mammalian species which often exhibit seasonal movements in resource and habitat utilization patterns. (Gupta and Krishnamurthy 2023; Gautam et al. 2019; Mallegowda et al. 2015)	MODIS
NDVI of summer	Spring (March–May), Summer (June–August), Post‐monsoon (September–November) and Winter (December–February)	—		
NDVI of winter		—		
NDVI of post‐monsoon		—		
Barren land	Land cover types at 500 m resolution (MCD12Q1, version 051, year 2010)	%	Different land cover types provide varying levels of shelter and resources, influencing the distribution and movement patterns of mammals (Zungu et al. 2020; Pillay et al. 2011).	
Broadleaf forest (BDLF)		%		
Mixed forest (MF)		%		
Grasslands		%		
Needle leaf forest (NDLF)		%		
Group 3: Topographic				
Elevation (DEM)	SRTM elevation data at 90 m resolution	m	Elevation affects climate, vegetation types, and food availability, making it an essential variable for modeling species distributions (Pal et al. 2021; Karanth et al. 2009)	CGIAR‐CSI
Topographical Positional Index (TPI)	Calculated based on the elevation data using the Geomorphometry and Gradient Metrix Toolbox in ArcGIS (Evans et al. 2014)	—	TPI helps in identifying the relative position of a pixel concerning surrounding landscape, indicating potential ecological niches, such as ridges, valleys, or slopes, that might be more favorable for species (Sultaire et al. 2023; Olson et al. 2017)	
Terrain ruggedness Index (TRI)		—	Terrain ruggedness, is crucial for understanding habitat heterogeneity; rugged landscapes offer shelter, food, and movement opportunities for various mammal species (Bandyopadhyay et al. 2022; Pal et al. 2021)	
Compound topographic index (CTI)		—	It reflects the potential for water accumulation and drainage patterns which are critical for many species, especially during dry seasons or in arid regions (Brncic et al. 2015; Rabanal et al. 2010)	
Water bodies	Land cover type at 500 m resolution (MCD12Q1, version 051, year 2010)	%	Water bodies serve as both drinking sources and areas for cooling and foraging, influencing movement patterns, migration routes, and territory establishment (Behera et al. 2024; Rondinini et al. 2011)	MODIS
Group 4: Human influences				
Combined class of croplands or agricultural land and cropland/natural vegetation mosaic	Land cover type at 500 m resolution (MCD12Q1, version 051, year 2010)	%	The interaction between natural habitats and agricultural land can influence mammal movement patterns and distribution, with agriculture land serving as a barrier or corridor depending on its location and management (Pillay et al. 2011; Karanth et al. 2009)	MODIS
Global human footprint (HFP)	Anthropogenic impacts on the environment for the period 1995–2004, Last of the Wild Data Version 2, 2005	%	It provides a comprehensive measure of human influence on the environment, crucial for understanding the impact of human activities on large mammal species. The dataset including population density, built‐up areas, roads, railroads, and navigable rivers, represents critical factors influencing habitat quality and availability for mammals (Allan et al. 2019; Venter et al. 2016)	Last of the Wild, v2 (http://sedac.ciesin.columbia.edu/wildareas/)
Urban and built‐up	Land cover type at 500 m resolution (MCD12Q1, version 051, year 2010)	%	These areas indicate regions of human development, which often lead to habitat fragmentation, poaching and road mortality. It allow to assess how these developments influence mammal distributions and habitat suitability, particularly for species sensitive to human disturbance (Tyagi et al. 2024; Marion et al. 2024)	MODIS
Group 5. Landscape Metrics				
Aggregation index (AI)	It is used to assess the degree of aggregation or clumping of patches within a landscape. It measures the tendency of similar land cover or land use categories to group together		It quantifies the degree of spatial aggregation of similar land cover types, indicating the extent of habitat fragmentation. High AI values suggest larger, more contiguous habitat patches, which are essential for species that require expansive, uninterrupted territories for foraging, breeding, and movement (Mukherjee et al. 2019)	FRAGSTAT v 4.2
Patch Area Distribution (AREA_AM)	Quantifies the distribution of patch sizes within a specific land cover or land use class in a landscape		This measure helps assess the availability of suitable habitats for species, whose movement and survival often depend on habitat size. Larger, more contiguous patches typically support more stable populations, providing ample resources such as food and shelter, which are essential for species	
Patch Cohesion Index (COHESION)	Measures the spatial connectedness of patches belonging to the same land cover or land use category within a landscape		It quantifies how well patches of the same habitat type are connected, which is crucial for species that require large, continuous areas for movement, foraging, and breeding (Sarkar et al. 2018; Nandy et al. 2020)	
Contagion (CONTAG)	Measures the extent to which patch types are aggregated or clumped. It is inversely related to edge density		It quantifies the spatial interspersion and adjacency of different land cover types, particularly focusing on habitat fragmentation and patch distribution (Rath et al. 2022; Nagendra et al. 2006)	
Edge Density (ED)	Quantifies the length of edges or boundaries between different land cover and land use classes in a landscape relative to the total landscape area		It quantifies the amount of edge relative to the total area of the landscape, typically measured as the total length of habitat edges per unit area (Mukherjee et al. 2019; Kumar et al. 2018)	
Radius of Gyration Distribution (GYRATE_AM)	Characterizes the spatial distribution of patch centroid locations within a landscape class		It is a landscape metric used to measure the spatial arrangement of habitat patches by evaluating the distribution of area around the centroid of each patch. It quantifies the extent to which patches are spread out relative to their center. This metric provides insights into habitat fragmentation and the availability of contiguous habitats, as species often rely on connected, expansive areas for movement, foraging, and reproduction. A larger radius of gyration typically indicates larger, more cohesive habitat patches, which are generally more suitable for large mammal populations (Sarkar et al. 2018; Carvalho et al. 2009)	
Largest Patch Index (LPI)	Quantifies the percentage of total landscape area comprised by the largest patch. As such, it is a simple measure of dominance		This index provides insights into habitat fragmentation, with a higher LPI indicating a more contiguous and less fragmented landscape, which is often beneficial for species that require large, continuous habitats for movement, foraging, and reproduction (Mukherjee et al. 2019; Sarkar et al. 2018)	
Patch Density (PD)	Quantify the number of patches of a specific landscape class within a landscape		Higher PD values indicate a more fragmented landscape with numerous smaller patches, while lower values suggest a more continuous habitat. Higher PD can be detrimental, as fragmented landscapes may impede movement, reduce access to resources, and increase the risk of inbreeding (Mukherjee et al. 2019; Sarkar et al. 2018)	

### 3.3 Multi‐Scale Data Processing

Six geographical scales—250, 500, 1000, 2000, 4000, and 8000 m radii—were used to estimate the focal mean of each variable around every presence site for the selected species, following the approach described by Mateo‐Sanchez et al. (2014). This analysis applied these scales to search neighborhoods using a moving window analysis with the focal statistics tool in ArcGIS 10.3. The forest cover layer was used to compute landscape indices, with the moving window analysis performed in FRAGSTATS v4.2 (McGarigal et al. 2012). Eight landscape‐level metrics were derived, including four indices from Area and Edge metrics (Patch Area Distribution, Edge Density, Radius of Gyration Distribution, and Largest Patch Index) and four from Aggregation metrics (Aggregation Index, Patch Cohesion Index, Contagion, and Patch Density). Output raster layers were generated for each variable at each scale across the study area to extract the focal mean at each location.

### 3.4 Multi‐Scale Optimized Univariate Modeling

We assessed the predictive strength of all 57 variables using the MaxEnt jackknife test for Area Under the Curve (AUC). For each environmental variable, we identified the scale that achieved the highest test AUC in a univariate model and incorporated it into the species’ multivariate models. This process resulted in ‘full models,’ where all variables were used at their best‐performing scale. Environmental variables with test AUC values of 0.6 or lower (Shabani et al. 2018) were excluded from the analysis. The remaining variables were checked for multicollinearity (correlation coefficient, *r* ≥ 0.6) using the ‘corrr’ package (Dormann et al. 2013). The number of variables retained for each species varied, with 8 variables for the northern red muntjac, 10 for the common palm civet, 7 for the dhole, 6 for the Asiatic golden cat, 8 for the Indian wild pig, 12 for the leopard cat, 11 for the large Indian civet, 10 for the masked palm civet, 6 for the serow, and 11 for the yellow‐throated marten. These retained variables were subsequently used for further analysis to model the habitat suitability of each species.

### 3.5 Multivariate Modeling and Model Validation

An ensemble modeling approach was adopted to enhance prediction accuracy and reduce uncertainty, forecasting habitat suitability for all 10 species (Donati et al. 2022; Bolliger et al. 2017) using the ‘biomod2’ package in R‐v3.5.1 (Thuiller et al. 2009, 2016; Hao et al. 2019). Ensemble modeling provides more reliable results than individual models, with lower mean errors and less uncertainty in predictions (Araújo and New 2007). The study utilized two regression‐based models, the Generalized Linear Model (GLM) and the Generalized Additive Model (GAM), alongside a machine‐learning model, Maximum Entropy (MaxEnt). For each algorithm, five sets of pseudo‐absences were generated, with each set containing 10,000 locations (Barbet‐Massin et al. 2012). To assess the accuracy of the models, a repeated data‐splitting procedure (cross‐validation) was used. The data, consisting of occurrence records and each pseudo‐absence dataset, was randomly split into a training set (80%) for calibration and a testing set (20%). In combination, this process resulted in a total of 75 models (3 niche‐based algorithms × 5 cross‐validations × 5 pseudo‐absence samples; Tran et al. 2023; Thuiller et al. 2016). The importance of each environmental factor was evaluated for each model, and for each split sample, five permutation tests were run using the built‐in function in the *biomod2* package (Thuiller et al. 2016). In these tests, each environmental factor was randomly shuffled, and the model's performance was re‐evaluated. The change in performance was measured by comparing the original prediction to the prediction made with the shuffled data. This difference was calculated as one minus the correlation score between the original and shuffled predictions (Thuiller et al. 2014). The importance score ranged from 0 to 1, with 1 indicating that the factor had the highest influence on the model's predictions.

Model accuracy was assessed using the AUC of the receiver operating characteristic (ROC; Allouche et al. 2006). For each species, we assessed and compared the performance of the habitat suitability models, ensuring that models with an AUC < 0.9 were considered as weights. Subsequently, we classified the predicted area into four equal intervals of suitable range for the species: not suitable (< 0.25), low (0.25–0.5), moderate (0.5–0.75), and high (> 0.75) relative occurrence probability zones. However, it is important to note that relative occurrence probability only indicates where the species is most likely to occur. It does not provide information on whether the best habitat contains the species in 90% of the samples or just 10% (Merow et al. 2013; Khan et al. 2020). Using *biomod2*, we calculated the contribution of each variable for every species across all models. Additionally, we generated and interpreted response curves illustrating the relationship between the presence points and the most influential variables in each model for all 10 species.

### 3.6 Species Richness

Species richness was calculated by summing the predicted habitat suitability values for all 10 species at each pixel in the study area, estimating the potential number of species supported in each location. For each pixel, the richness value represented the total number of species predicted to be suitable if multiple species were viable. The area with a specific richness level (e.g., for 1, 2, or 3 species) was determined by summing the total area where the richness value matched each species count inside and outside Protected Areas (PAs). This is calculated as:

$$\text{Area with species richness}=\sum \left(\text{Area of Pixels with Specific Richness}\right)$$

The percentage of richness for each level (e.g., 1 species, 2 species, etc.) inside or outside PAs was calculated by dividing the area of richness inside or outside PAs by the total area of that specific richness level across both inside and outside PAs, then multiplying by 100:

$$\text{Percentage of Richness Inside or outside}\;\mathrm{PAs}=\left(\frac{\text{Area with Specific Richness Inside or outside}\;\mathrm{PAs}}{\text{Total Area of Species Richness Inside or outside}\;\mathrm{PAs}}\right)*100$$

The overall species richness inside and outside PAs was determined by summing the total area of species richness across all levels and dividing it by the total area of species richness across both inside and outside PAs. The formula for the percentage of overall richness inside PAs is:

$$\text{Percentage of Overall Richness Inside or outside}\;\mathrm{PAs}=\left(\frac{\text{Total Area of Richness Inside or outside}\;\mathrm{PAs}}{\text{Total Area of Inside}\;\mathrm{PAs}\;\text{or Outside}\;\mathrm{PAs}}\right)*100$$

## 4 Result

### 4.1 Univariate Scaling

**FIGURE 3:**
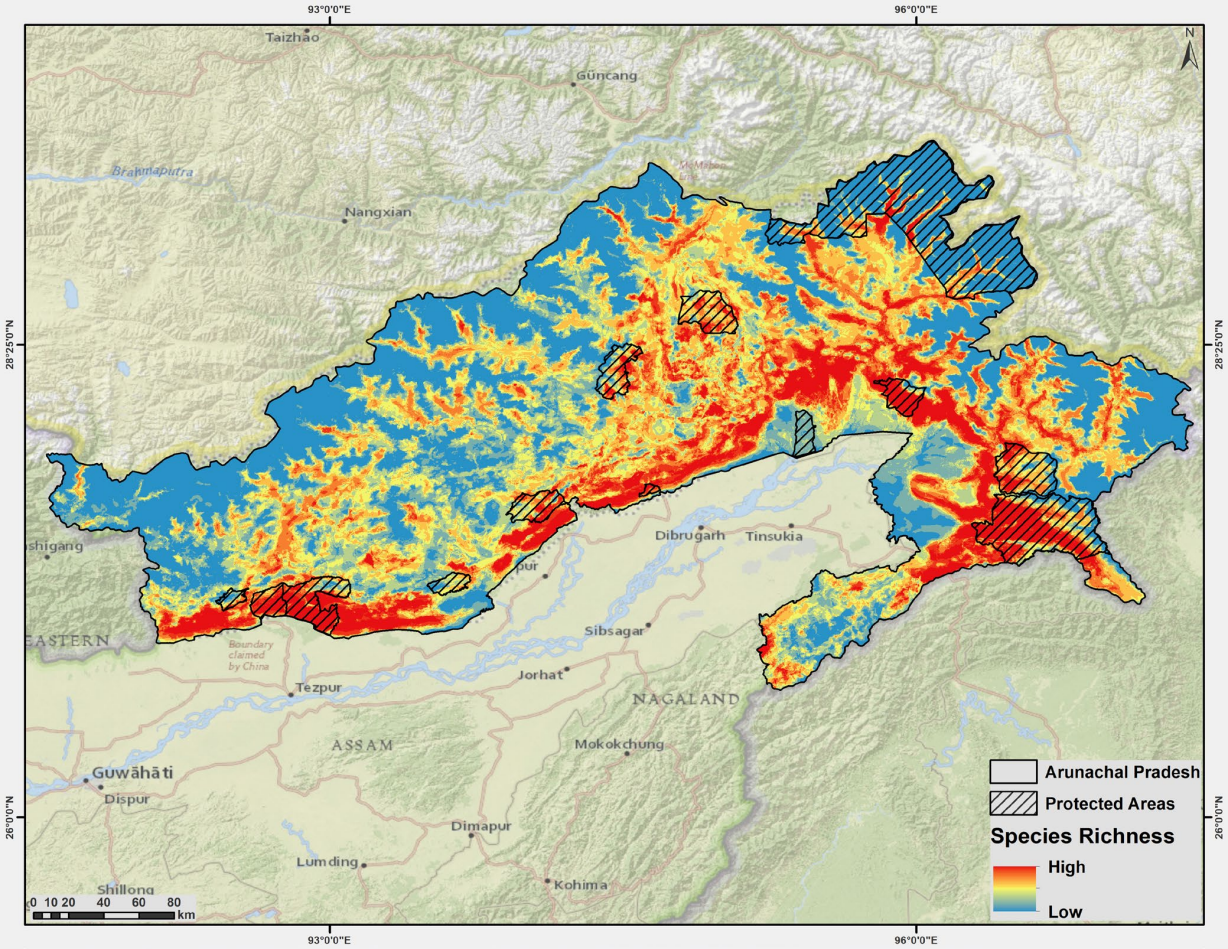
Predicted species richness (10 modelled species) across Arunachal Pradesh, India.

A total of six spatial scales (250–8000 m) for each predictor variable were chosen for univariate modeling. Overall, the scales at a broader spatial extent (8000 m) had the highest selection frequency (Figure 3). Ungulates showed a strong relationship for predictor variables at medium to broader scales, whereas carnivores selected variables more frequently at a broader scale.

### 4.2 Multivariate Modeling and Variable Importance

BIOMOD employs a randomization procedure to determine the importance of each variable. Variable contributions and response curve plots for the selected and scaled variables are available in Appendix S2, S3. Elevation was the primary predictor variable influencing carnivores, demonstrating significant importance across all carnivore species. For dhole, key predictor variables included broadleaf forest, population, and post‐monsoon at a large spatial scale (4000–8000 m), elevation and precipitation seasonality (Bio 15) at medium scales (2000 m), and radius of gyration distribution at fine scales (500 m). Elevation, Bio 15, human population, and summer exhibited predictor variable mean values exceeding 0.5, signifying their importance in predicting dhole occurrence. In the case of the Asiatic golden cat, three variables (mixed forest, contagion, and potential evapotranspiration wet quarter) out of six predictor variables were selected at the broadest scale (8000 m), elevation was chosen at a medium scale, and two predictive variables (population and compound topographic index) were selected at a fine scale. Elevation (0.65_mean_ ± 0.06_SE_) and potential evapotranspiration wet quarter (0.51_mean_ ± 0.12_SE_) were identified as critical variables influencing the habitat associations of the Asiatic golden cat. Yellow‐throated marten's habitat was linked to five predictor variables (broadleaf forest, mixed forest, contagion, edge density, and largest patch index) at the broadest scale (8000 m). Additionally, four variables (Bio 15, grassland, winter, and human footprint) were chosen at a medium scale (1000–2000 m), while two predictor variables (elevation and post‐monsoon) were selected at a fine scale (250 m). Elevation (0.45_mean_ ± 0.08_SE_), mixed forest (0.23_mean_ ± 0.05_SE_), and broadleaf forest (0.25_mean_ ± 0.11 _SE_ ) played pivotal roles in predicting the distribution of the yellow‐throated marten. For the leopard cat, nine predictor variables were selected at large scales (4000–8000 m), summer was chosen at a medium scale (1000 m), and two predictor variables (elevation and post‐monsoon) were selected at fine scales (250–500 m). Elevation (0.67_mean_ ± 0.15_SE_) emerged as the most significant predictor variable contributing to leopard cat occurrence.

In herbivores, the Northern red muntjac exhibited four key predictor variables linked to its habitat selection. Agricultural land, broadleaf forest, built‐up areas, and human population were selected at the large scale (8000 m), while post‐monsoon and mean summer were chosen at the medium scale (1000–2000 m). Elevation played a crucial role at a fine scale (250 m) in determining the Northern red muntjac's presence, with elevation and human population being the most influential predictors. For the mainland serow, broadleaf forest and cohesion index were key predictors at the large scale (8000 m), Bio15 at the medium scale, and elevation, human population, and post‐monsoon at the fine scale. Elevation and human population were the most influential factors in determining its presence. Indian wild pig habitat selection was influenced by multiple factors. At the large scale, broadleaf forest, grassland, and patch density were key predictors, while Bio15 and human population played crucial roles at the medium scale (1000–2000 m). At the fine scale (250–500 m), elevation, winter, and potential evapotranspiration (wet quarter) were the primary determinants. Elevation was the most significant factor influencing its presence.

Among civets, masked palm civets exhibited five significant predictor variables linked to their habitat preferences. These included mixed forest, winter, human population, Bio15, and grassland, which were selected at a large scale (8000 m). At the medium scale (2000 m), post‐monsoon and contagion index were identified as essential predictor variables. In contrast, at the fine scale (500 m), three predictor variables played a pivotal role: broadleaf forest, elevation, and human footprint. Notably, elevation (0.42_mean_ ± 0.12_SE_) and human population (0.42_mean_ ± 0.09_SE_) emerged as the most influential variables influencing the presence of masked palm civets. For large Indian civets, nine predictor variables were identified at the large scale (4000–8000 m), while grassland and the terrain ruggedness index were selected at the medium (2000 m) and fine scale (500 m), respectively. The compound topographical index (0.57_mean_ ± 0.09_SE_) and elevation (0.32_mean_ ± 0.14_SE_) were the primary contributing factors influencing the presence of large Indian civets. In the case of common palm civets, six predictor variables were selected at a large scale. Elevation, patch density index, Bio 15, and Potential evapotranspiration wet quarter were chosen at the fine scale (500 m). The distribution of the common palm civet was predominantly influenced by broadleaf forest (0.41_mean_ ± 0.12_SE_) and elevation (0.42 ± 0.14_SE_), establishing them as the foremost significant predictor variables.

### 4.3 Comparison of Suitable Habitat Areas for Species Inside and Outside Protected Areas (PAs)

Based on a study area of 83,743 km^2^ in Arunachal Pradesh, with PAs covering 9795.99 km^2^ and areas outside PAs covering 73,947.01 km^2^, the comparison of suitable areas for species inside and outside PAs presented in Table 3 reveals distinct patterns of habitat distribution. The Northern Red Muntjac has 1456.81 km^2^ (5.32%) of high‐suitability habitat outside PAs and 757.81 km^2^ (7.48%) within PAs, indicating a higher concentration of suitable habitat inside PAs. The Common Palm Civet shows 1054.44 km^2^ (3.85%) of high‐suitability habitat outside PAs and 630.43 km^2^ (6.23%) within PAs, with a larger proportion inside PAs. Similarly, the dhole has 1011.69 km^2^ (3.70%) outside PAs and 742.37 km^2^ (7.33%) inside PAs. The Leopard Cat displays 1128.18 km^2^ (4.12%) outside PAs and 723.75 km^2^ (7.15%) within PAs, suggesting that it is more suited to PAs. The Large Indian Civet has 1779.38 km^2^ (6.50%) outside PAs and 451.37 km^2^ (4.46%) inside PAs, with a significant portion of its suitable habitat outside PAs. The Masked Palm Civet shows 583.56 km^2^ (2.13%) of high‐suitability habitat outside PAs and 267.69 km^2^ (2.64%) within PAs, with a relatively larger proportion outside PAs. The Asiatic Golden Cat has 921.75 km^2^ (3.37%) outside PAs and 605.81 km^2^ (5.98%) inside PAs, indicating that it prefers areas within PAs. The Mainland Serow has 7019.18 km^2^ (25.64%) outside PAs and 1309.69 km^2^ (12.93%) within PAs, with its primary suitable habitat found outside PAs. The Indian Wild Pig has 8053 km^2^ (29.41%) outside PAs and 1067.75 km^2^ (10.54%) within PAs, showing a greater preference for habitats outside PAs. Lastly, the Yellow‐throated Marten has 4369.93 km^2^ (15.96%) outside PAs and 1301.38 km^2^ (12.85%) within PAs, with a higher proportion of its suitable habitat found outside PAs.

**TABLE 3:** Suitable habitat area (km^2^ and %) for species, categorized by low, medium, and high suitability both inside and outside PAs.

Species	Suitable area outside PAs				Suitable area inside PAs			
	Low (km^2^, %)	Medium (km^2^, %)	High (km^2^, %)	Total suitable area (km^2^, %)	Low (km^2^, %)	Medium (km^2^, %)	High (km^2^, %)	Total suitable area (km^2^, %)
Northern‐red Muntjac	13,175.68 (13.74)	8266.81 (14.59)	1456.81 (5.32)	22,899.3 (12.72)	1888.75 (18.65)	1690.12 (16.69)	757.81 (7.48)	4336.68 (14.2)
Common Palm Civet	7229.63 (7.54)	2842.06 (5.02)	1054.44 (3.85)	11,126.13 (6.18)	808.62 (7.98)	591.62 (5.84)	630.43 (6.23)	2030.67 (6.64)
Dhole	11,025.88 (11.50)	4513.68 (7.96)	1011.69 (3.70)	16,551.25 (9.2)	1483.62 (14.65)	1057 (10.44)	742.37 (7.33)	3282.99 (10.75)
Leopard Cat	6128.18 (6.39)	2721.31 (4.8)	1128.18 (4.12)	9977.67 (5.54)	836.5 (8.26)	708.56 (7)	723.75 (7.15)	2268.81 (7.42)
Large Indian Civet	3222.19 (3.36)	1764.57 (3.11)	1779.38 (6.50)	6766.14 (3.76)	665.31 (6.57)	396.12 (3.91)	451.37 (4.46)	1512.8 (4.95)
Masked Palm Civet	3015.18 (3.14)	1131.75 (2)	583.56 (2.13)	4730.49 (2.62)	613 (6.05)	344.44 (3.40)	267.69 (2.64)	1225.13 (4.01)
Asiatic Golden Cat	3328.44 (3.47)	1292 (2.28)	921.75 (3.37)	5542.19 (3.08)	617.37 (6.10)	457.44 (4.52)	605.81 (5.98)	1680.62 (5.5)
Mainland Serow	13,794.44 (14.39)	11,587.07 (20.45)	7019.18 (25.64)	32,400.69 (18.01)	1834.75 (18.12)	1613.12 (15.93)	1309.69 (12.93)	4757.56 (15.57)
Indian Wild Pig	16,382.75 (17.09)	12,936.62 (22.83)	8053 (29.41)	37,372.37 (20.77)	1915.25 (18.91)	1715.31 (16.94)	1067.75 (10.54)	4698.31 (15.38)
Yellow‐throated Marten	18,583.44 (19.38)	9615 (16.97)	4369.93 (15.96)	32,568.37 (18.10)	1746.25 (17.24)	1697.44 (16.76)	1301.38 (12.85)	4745.07 (15.53)
Total Area				73,947.01				9795.99

*Note:* The table also includes overall totals for habitat area and corresponding percentages, summarizing the distribution across PA classifications. Percentages indicate the proportion of the total area occupied by each species in each category.

### 4.4 Species Richness Inside and Outside of PAs

The analysis indicates that a lower percentage of the landscape within PAs (10.95%) supports a species richness value of 1 compared to areas outside of PAs (15.81%). For species richness value 2, 8.60% of the landscape within PAs supports this level versus 16.95% outside, and for value 3, 11.85% inside contrasts with 18.79% outside. This trend continues for species richness value 4, with 14.98% of the landscape within PAs supporting it compared to 19.18% outside. However, a reversal occurs for higher richness values; for example, species richness value 5 appears in 14.40% of the landscape within PAs as opposed to 12.90% outside, while species richness value 6 shows 11.55% inside versus 7.57% outside. An even more pronounced shift is evident for species richness values 7 through 10, where 11.88%, 8.10%, 4.96%, and 2.72% of the landscape within PAs support these values compared to 4.49%, 2.73%, 1.33%, and 0.25% outside, respectively. In terms of total richness, although higher richness values occur more frequently inside PAs, the cumulative species richness remains greater outside (72.63%) than within PAs (61.90%; Table 4).

**TABLE 4:** Species richness (km^2^ and %) inside and outside PAs in Arunachal Pradesh for up to 10 species.

Species richness	Inside PA (km^2^, %)	Outside PA (km^2^, %)
1	660.94 (10.95)	7950 (15.81)
2	518.94 (8.60)	8524.37 (16.95)
3	714.94 (11.85)	9446.18 (18.79)
4	903.75 (14.98)	9642.25 (19.18)
5	869.06 (14.4)	6487.81 (12.90)
6	697 (11.55)	3808.37 (7.57)
7	716.56 (11.88)	2255.12 (4.49)
8	488.87 (8.10)	1371.18 (2.73)
9	299.44 (4.96)	670.5 (1.33)
10	163.87 (2.72)	124.12 (0.25)
Total richness	6033.37 (61.90)	50,279.93 (72.63)

*Note:* The proportion of area within and outside of PAs supporting variable numbers of species investigated in this study is shown.

### 4.5 Habitat Suitability and Predicted Distribution Range of Species

**FIGURE 4:**
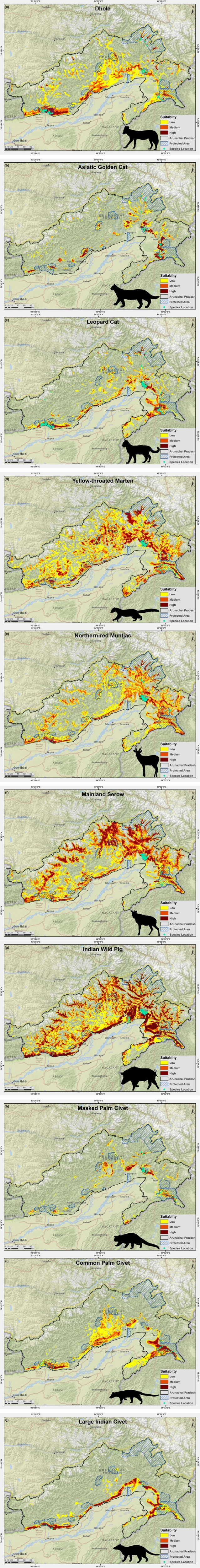
Predicted habitat suitability of a) Dhole, b) Asiatic golden cat, c) Leopard cat, d) Yellow‐throated marten, e) Northern‐red muntjac, f) Mainland serow, g) Indian wild pig, h) Masked palm civet, i) Common palm civet, and j) Large Indian civet.

The predicted model identified the southern part of the study area as the primary concentration of suitable habitats for carnivores. However, the yellow‐throated marten is found more widely across the study area. Among these carnivores, the dhole, leopard cat, and yellow‐throated marten preferred elevated regions and broadleaf forest habitats, while the Asiatic golden cat favored mixed forest habitats. Notably, all carnivore species actively avoided areas inhabited by humans (Figure 4a–d).

Among the herbivores, all species exhibited a strong preference for broadleaf forest habitats that spanned the entire study area. Northern red‐muntjac and Mainland serow favored higher elevations with low levels of human disturbance, as illustrated in Figure 4e,f. However, the Indian wild pig displayed a unique affinity for grassland areas and degraded habitat patches near human settlements (Figure 4g). As for civets, their primary habitat preference was in the lower elevated forest regions along the southern boundary of the study area, as depicted in Figure 4h–j.

## 5 Discussion

This study examined habitat suitability patterns for 10 mammalian species in Arunachal Pradesh using a multi‐scale ensemble modeling approach. The results highlighted the influence of environmental and anthropogenic variables on species distributions, emphasizing the roles of forest cover, climatic variability, topographic, and human disturbance. Although forested areas within PAs provided significant suitable habitats, large portions of high‐suitability areas also existed outside PAs. The multi‐scale approach provided deeper insights into how species responded to different spatial extents, highlighting the necessity of evaluating environmental variables across multiple scales.

The association between forest cover and habitat suitability highlights the critical role of intact forests in supporting selected mammalian species. Forest‐related variables consistently demonstrated a positive effect on habitat suitability for the studied species, emphasizing the importance of preserving forest ecosystems, which serve as critical habitats for native wildlife in Arunachal Pradesh. Similar patterns were observed by Ahmad et al. (2023) in the region, who reported a strong positive affinity of 27 mammal species for forest cover. Broadleaf and mixed forests were among the most influential predictors, likely due to their structural complexity, food availability, and microclimatic stability (Salom‐Pérez et al. 2021). These forests provide essential cover, forage, and shelter, supporting the persistence of these mammalian species. Species richness was generally higher in contiguous forested landscapes, emphasizing the importance of habitat continuity for biodiversity conservation.

The comparison of suitable habitats inside and outside PAs in Arunachal Pradesh highlights distinct patterns in species distribution, reflecting variations in habitat preference, ecological requirements, and human influences. Certain species, such as the Northern‐red Muntjac, dhole, leopard cat, and Asiatic Golden Cat, show a higher proportion of suitable habitat within PAs, likely due to better forest cover, legal protection from hunting, and prey availability (Penjor, Kaszta, et al. 2021). These species often rely on undisturbed ecosystems, which are more prevalent within PAs. In contrast, species like the Large Indian Civet, Masked Palm Civet, Mainland Serow, and Indian Wild Pig have a significant proportion of suitable habitat outside PAs, which can be attributed to their adaptability to human‐modified landscapes, broader ecological tolerance, and the availability of food resources in agricultural areas and secondary forests (Punjabi and Rao 2017). The presence of suitable habitats outside PAs underscores the importance of conservation strategies beyond protected zones, including habitat corridors, sustainable land‐use planning, and community‐based conservation initiatives to ensure species persistence in both protected and non‐protected landscapes. Strengthening protection within PAs while promoting coexistence in human‐dominated areas is crucial for maintaining biodiversity in the region (Pringle 2017; D'Aloia et al. 2019).

This aligns with previous studies demonstrating the ecological significance of undisturbed forests in maintaining habitat connectivity, reducing fragmentation effects, and ensuring resource availability for wildlife (DeFries et al. 2010; Srivastava and Tyagi 2016). Species in fragmented landscapes exhibit diverse adaptations to their environments, with habitat preferences influenced by a combination of forest connectivity, prey abundance, climatic factors, and human disturbances. Species dependent on contiguous forests for hunting and shelter, such as many carnivores, rely on expansive closed‐canopy habitats to maintain stable populations (Wangmo et al. 2020; Suraci et al. 2020). Elevation plays a crucial role, with mid‐to‐high elevations offering reduced competition and lower human disturbances, whereas lower elevations often provide greater prey densities but are more susceptible to habitat fragmentation (Kamler et al. 2020; Decœur et al. 2023). Mammals in Arunachal Pradesh exhibit diverse habitat preferences, influenced by a combination of forest structure, topographic, climate, and anthropogenic pressures. While many species demonstrate a strong preference for dense forests, their distributions are also shaped by climatic and topographical variables. Elevation is an important factor in habitat use, with mid‐elevation zones providing optimal conditions for several species that rely on a balance of prey availability, reduced competition, and fewer human disturbances (Kamler et al. 2020; Decœur et al. 2023). Species exhibit a preference for higher elevations, likely due to reduced anthropogenic pressures and more stable habitat conditions (Zeng et al. 2018; Petersen et al. 2021; Ahmad and Gopi 2024). Conversely, species that occupy lower elevations show adaptability to fragmented landscapes but also experience greater vulnerability to human activities, such as agricultural expansion and habitat conversion (Lyngdoh et al. 2014; Sunarto et al. 2015; Graham et al. 2014; Choki et al. 2023).

Seasonal climatic factors played a significant role in shaping habitat suitability. Precipitation seasonality (Bio 15) and potential evapotranspiration during wetter periods were key predictors across spatial scales (Deng et al. 2022). These variables influence prey dynamics, vegetation growth, and water availability, making them crucial determinants of habitat preference. Regions with stable precipitation regimes were associated with higher suitability, highlighting the importance of consistent water availability for sustaining mammalian populations (Torres‐Romero and Olalla‐Tárraga 2015; Smith 2012). Moreover, post‐monsoon conditions were observed to influence habitat suitability, as they determine vegetation regrowth and prey abundance, which are critical factors for both carnivores and herbivores. Changes in precipitation can significantly alter ecosystem productivity, affecting both prey availability and the structural integrity of forests that serve as critical habitats for these species (Ferretti and Fattorini 2020).

Multi‐scale species‐habitat modeling in this study revealed a general preference for PAs and forest cover, with a pronounced avoidance of anthropogenic variables. These associations were most evident at broader spatial scales (4000–8000 m; Joshi et al. 2022; Bharali and Khan 2011), underscoring the necessity of conserving large forest landscapes under protection. The strong negative correlation with anthropogenic factors, detectable at a broad scale in our models, indicates that human activities have ecological effects that extend well beyond their immediate footprint (Penjor, Wangdi, et al. 2021). The impacts of deforestation, agricultural expansion, and human settlement are not limited to direct habitat loss but also lead to fragmentation, edge effects, and increased human‐wildlife conflicts, all of which contribute to declining habitat suitability.

Species in fragmented landscapes exhibit a range of adaptations, with some thriving in lower elevations due to higher prey densities, while others depend on mid‐to‐high elevations where human disturbances are lower. Key habitat features such as forest connectivity, prey abundance, and vegetation structure play crucial roles in shaping distributions. However, human‐wildlife conflict is a persistent issue in agricultural landscapes, as species are often drawn to farmlands in search of food, increasing interactions with local communities (Gulati et al. 2021). Effective conservation strategies must focus on minimizing fragmentation, enhancing habitat corridors, and promoting sustainable land‐use practices to mitigate these conflicts (Ballari and Barrios‐García 2014). Agricultural expansion emerged as a major driver of reduced habitat suitability, as it contributes to habitat fragmentation and an increased vulnerability to human‐wildlife conflict. While agriculture can provide high‐quality forage for herbivores, it frequently exacerbates conflicts, particularly in areas where carnivores are present. Carnivores are highly susceptible to these pressures, as they require large, undisturbed habitats for hunting and reproduction (Woodroffe et al. 2005; Gopi et al. 2010; Rostro‐García et al. 2016; Penjor et al. 2022). In agricultural‐village mosaics, carnivores often predate on livestock, leading to retaliatory killings and further population declines. Intermediate levels of agricultural mosaics may sometimes enhance habitat diversity and attract certain species, but extensive fragmentation typically disrupts ecosystems and places additional pressures on wildlife populations (Cushman and McGarigal 2003). The presence of suitable habitats outside PAs highlights the need for landscape‐level conservation strategies that maintain connectivity between protected and non‐protected areas, ensuring that species can move across different habitats without experiencing significant ecological barriers (Mozelewski et al. 2022; Chung and Lee 2022). Habitat connectivity between PAs and surrounding areas is vital for sustaining populations and ensuring genetic exchange. However, infrastructure development, including dams and highways, accelerates habitat degradation, particularly in Sikkim and Arunachal, increasing species vulnerability. Mitigating these impacts and safeguarding in situ and ex situ habitats are crucial for effective conservation (Datta‐Roy 2019). Given that a significant portion of suitable habitat extends beyond PAs, it is crucial to recognize the conservation value of these landscapes. Enhancing habitat connectivity between PAs and surrounding areas is essential for maintaining species populations, facilitating genetic exchange, and ensuring the long‐term persistence of biodiversity.

Although this study did not incorporate future climate change modeling, the potential impact of climate change on species distribution is undeniable. Changes in temperature, precipitation patterns, and the frequency of extreme weather events are likely to alter habitat suitability across landscapes. Previous studies (Sarkar et al. 2024; Malakoutikhah et al. 2020) have highlighted that climate change can lead to range contractions, shifts to higher altitudes or latitudes, and even local extinctions, particularly for species with limited adaptive capacity. Given the importance of these factors, future research on species distribution should integrate climate change scenarios to better understand and predict how habitats and species ranges may evolve, especially in vulnerable regions like Arunachal Pradesh. Addressing this gap is critical for effective conservation planning and ensuring the long‐term survival of biodiversity in a changing climate. The 10 mammal species studied in this research exhibit diverse habitat preferences and ecological roles, including both carnivores and herbivores. Their distribution patterns reflect unique adaptations to the landscapes of Arunachal Pradesh, with some species favoring contiguous, undisturbed forests, while others display a degree of adaptability to fragmented habitats. However, species vary in their tolerance to habitat changes. Carnivores generally require expansive territories with minimal human disturbances to thrive (Barker et al. 2023). However, some species can persist in fragmented landscapes if sufficient prey and cover are available, allowing them to adapt to human‐altered environments (Mortelliti and Boitani 2008). Understanding these distribution patterns is essential for designing targeted conservation strategies that consider species‐specific needs while maintaining landscape connectivity.

SDMs are essential tools for identifying priority areas to enhance biodiversity conservation efforts (Penjor, Wangdi, et al. 2021; Condro et al. 2021). The results highlight the essential role of PAs in biodiversity conservation, particularly in regions with higher species richness. The trend of increasing species richness within PAs at higher richness levels underscores their effectiveness in preserving undisturbed habitats and reducing human pressures. By safeguarding species with specific habitat requirements, PAs provide stable and controlled environments that support long‐term species survival. However, a substantial proportion of species richness (72.63%) is also found outside PAs, emphasizing the importance of forested landscapes beyond protected boundaries (DeFries et al. 2010). These areas serve as vital ecological corridors, facilitating species movement and genetic exchange while supporting species adaptable to human‐modified environments (Srivastava and Tyagi 2016). This finding underscores the complementary roles of PAs and surrounding forested areas in conservation.

Another important consideration is how species interact with landscape heterogeneity. The role of terrain ruggedness, forest patch cohesion, and habitat aggregation in determining habitat suitability suggests that species distributions are shaped not only by absolute environmental conditions but also by habitat configuration (Chakravarthy and Ratnam 2015). Fragmented landscapes with isolated patches of forest may reduce dispersal opportunities and increase mortality risks, particularly for species with limited mobility or specific habitat requirements (Fahrig 2003; Mortelliti et al. 2010). Our comprehensive analysis of habitat suitability in Arunachal Pradesh provided both a detailed examination of key ecological drivers and practical recommendations for conservation management. By consolidating species‐specific observations into broader patterns, we offered a cohesive framework that could inform future biodiversity assessments and policy decisions. This integrative approach advanced our understanding of species–environment interactions and underscored the need for adaptive management strategies that are responsive to both ecological and anthropogenic changes (Lawler 2009; Williams and Brown 2016). The insights gained from our study are intended to support efforts aimed at preserving the unique biodiversity of the Eastern Himalayan region while promoting sustainable coexistence between human communities and wildlife.

## 6 Limitations, Conclusions, and Management Implications

The models in this study effectively capture the distribution patterns of the examined mammalian species. While the sample coverage was limited, the largely continuous environmental gradients and ecological processes in the study region help mitigate uncertainty, allowing the model to generate reliable habitat suitability predictions. Numerous studies support the assumption that key habitat relationships persist across such landscapes, providing additional evidence for the robustness of our model predictions. To enhance prediction robustness, we utilized Biomod2, a widely recognized ensemble modeling framework integrating multiple algorithms and implementing rigorous validation techniques (e.g., AUC). This approach increases confidence in extrapolating habitat suitability to regions without direct training data. This study represents the first multi‐scale, multi‐species ensemble modeling effort in Arunachal Pradesh, providing novel insights into the spatial distribution of key mammalian species in one of India's biodiversity hotspots. The results serve as a critical resource for conservation planning and habitat suitability assessments for other elusive and endangered species. By identifying key environmental variables and habitat preferences, these models provide a framework that can be applied to similar species with overlapping ecological requirements. This is particularly valuable for species with limited occurrence data, as modeled relationships help predict potential distributions beyond direct observations. Understanding the interplay between environmental and ecological factors across spatial scales is essential for forecasting threats posed by human activities and climate change. SDMs are robust tools for assessing potential occurrence areas and identifying key variables influencing species distributions, including climatic, landscape, vegetation, topographic, and anthropogenic factors. Recognizing the limitations of extrapolation, we recommend interpreting suitability maps as probabilistic estimates rather than definitive classifications. To mitigate biases in areas with no training data, targeted field surveys and independent dataset validations are advised. This proactive approach will refine model accuracy and support more informed conservation decisions.

Future research should expand data collection in under‐sampled regions and compile broader datasets, including historical records, to refine mammal distribution patterns. While many species prefer habitats within PAs, significant portions of their suitable habitats lie outside these boundaries, often overlapping with human‐dominated landscapes. This increases the potential for human‐wildlife conflict, highlighting the need for landscape‐level conservation strategies that integrate both protected and unprotected areas. Promoting sustainable land‐use practices and enhancing connectivity between PAs will help mitigate conflicts and safeguard critical habitats. These findings directly inform regionally tailored conservation initiatives aimed at preserving biodiversity amid climate and land‐use challenges. The comprehensive insights gained from this study provide a foundation for future research and adaptive conservation planning, ensuring the long‐term survival of mammalian species in Arunachal Pradesh.

## Author Contributions

**Arif Ahmad:** conceptualization (equal), data curation (lead), formal analysis (lead), methodology (equal), software (lead), validation (equal), visualization (lead), writing – original draft (lead), writing – review and editing (equal). **Govindan Veeraswami Gopi:** conceptualization (equal), methodology (equal), resources (lead), supervision (lead), validation (equal), writing – review and editing (equal).

## Conflicts of Interest

The authors declare no conflicts of interest.

## Supporting information


**Appendix S1.** Georeferenced data sources.

**Appendix S2.** Variable importance plot for all mammalian species.

**Appendix S3.** Variable importance response curve.

## Data Availability

A Georeferenced data source Appendix S1. A variable importance plot for all 10 mammalian species Appendix S2. A variable importance response curve Appendix S3. Access the species raster files and SDM code here: https://drive.google.com/file/d/1AJaUjhVPUCFwoaspZynDE1aLl9A3GDsL/view?usp=drive_link.
