# Supplementary material for: Understanding Multi‐Scale and Multi‐Species Habitat Selection by Mammals in the Eastern Himalayan Biodiversity Hotspot
Source: Ecol Evol. 2025 Apr 23;15(4):e71247. doi: 10.1002/ece3.71247 (PMC12015752; doi:10.1002/ece3.71247)
Supplement: Supplementary file 1 — Appendix S1. Georeferenced data sources. [file ECE3-15-e71247-s002.docx]

**Appendix S1: Georeferenced data sources**

Chakraborty, R., Chakraborty, P., Borah, J. & Mize, T. (2014). Mammals of D’Ering Wildlife Sanctuary: A baseline survey report. WWF-India report.

Chauhan, D. S., Singh, R., Mishra, S., Dadda, T., and Goyal S. P. (2006). Estimation of tiger population in an intensive study area of Pakke Tiger Reserve, Arunachal Pradesh, India. Wildlife Institute of India, Dehradun, India

Dasgupta, S., Sarkar, S., Kayrong, Deori, D., Dada, T., Kaul, R., Ranjitsinh, M.K. & Menon, V. (2010). Distribution and status of takin *(Budorcas taxicolor)* along the Tibetan, Myanmar and Bhutan borders of India. A report of the Wildlife Trust of India submitted to CEPF.

Datta, A. (1999). Small carnivores in two protected areas of Arunachal Pradesh. *Journal-Bombay Natural History Society*, 96(3), 399-404.

Datta, A., Naniwadekar, R., & Anand, M. O. (2008). Occurrence and conservation status of small carnivores in two protected areas in Arunachal Pradesh, north-east India. *Small Carnivore Conservation*, *39*, 1-10.

Jhala, Y.V., Qureshi, Q. and Nayak, A.K. (eds) 2020. Status of tigers, copredators and prey in India, 2018. National Tiger Conservation Authority, Government of India, New Delhi, and Wildlife Institute of India, Dehradun. ISBN No. 81-85496-50-1.

Jhala, Y.V., Qureshi, Q., Yadav, S.P. 2021. Status of leopards, co-predators, and megaherbivores in India, 2018. National Tiger Conservation Authority, Government of India, New Delhi, and Wildlife Institute of India, Dehradun. ISBN - 81-85496-56-0

Naniwadekar, R., Shukla, U., Viswanathan, A., & Datta, A. (2013). Records of small carnivores from in and around Namdapha Tiger Reserve, Arunachal Pradesh, India. *Small Carnivore Conservation*, 49, 1-8.

Singh, C. & D. Gupta (2021). First photographic record of Mishmi Takin Budorcus taxicolor taxicolor and Red Goral Nemorhaedus baileyi from Kamlang Tiger Reserve, Arunachal Pradesh, India. *Journal of Threatened Taxa* 13(8): 19149–19152. <https://doi.org/10.11609/jott.7059.13.8.19149-19152>

WII (2019). Wildlife conservation plan for the impact zone of Etalin HEP, Dibang Valley District, Arunachal Pradesh. Wildlife Institute of India, Dehradun. Technical Report TR No/2019/01.
