# Supplementary figures and images for: Understanding Multi‐Scale and Multi‐Species Habitat Selection by Mammals in the Eastern Himalayan Biodiversity Hotspot
Source: Ecol Evol. 2025 Apr 23;15(4):e71247. doi: 10.1002/ece3.71247 (PMC12015752; doi:10.1002/ece3.71247)

**Appendix S2-** **Variable importance plot for all mammalian species**


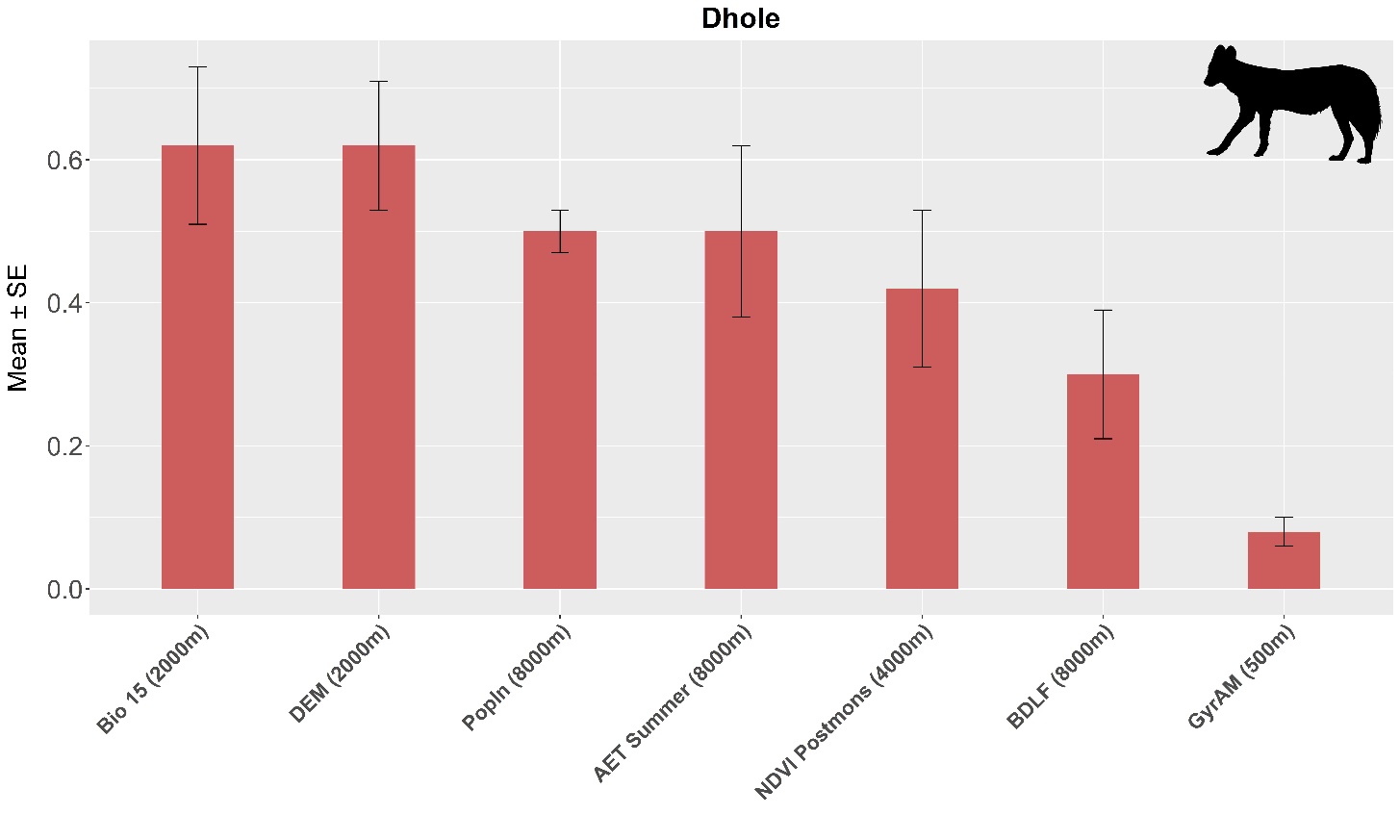

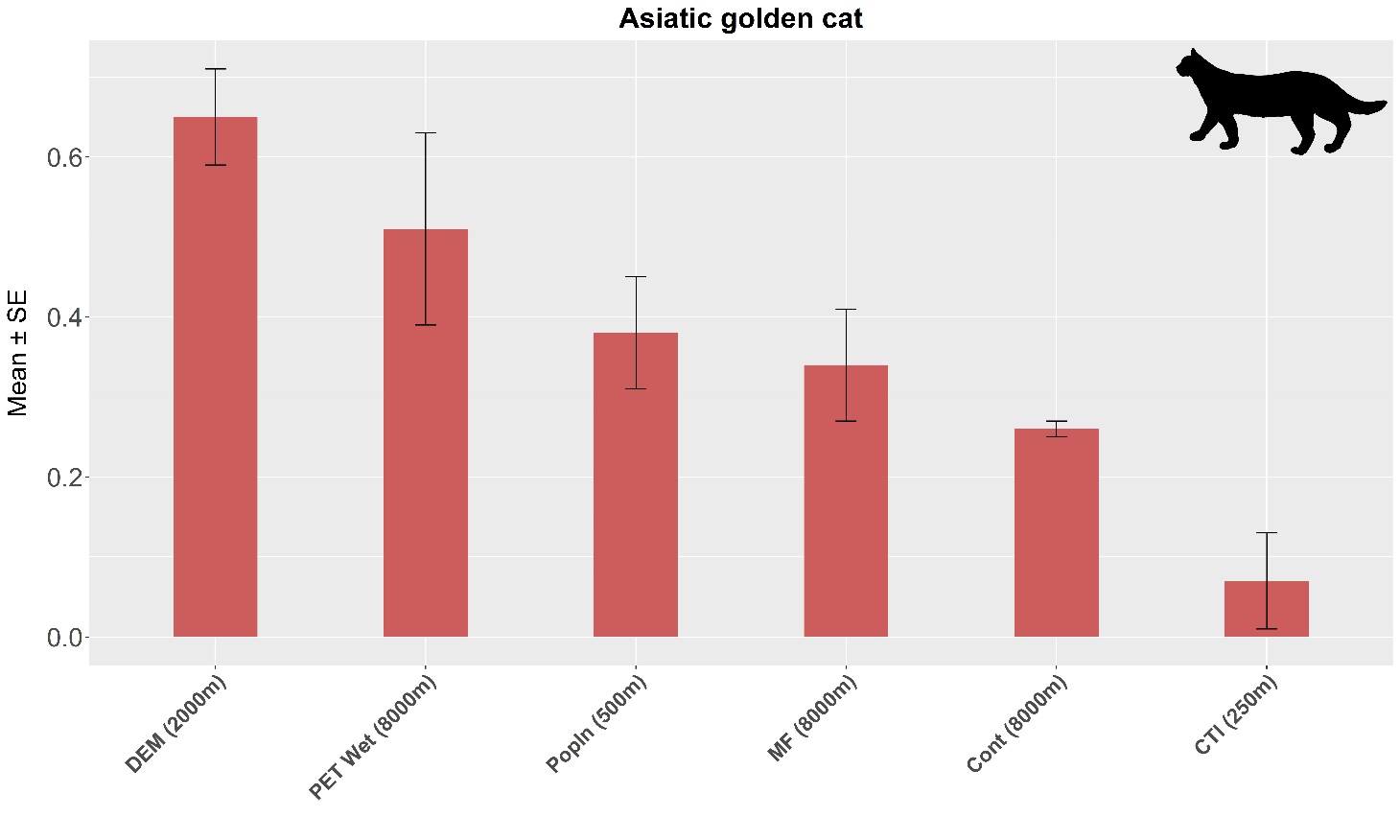

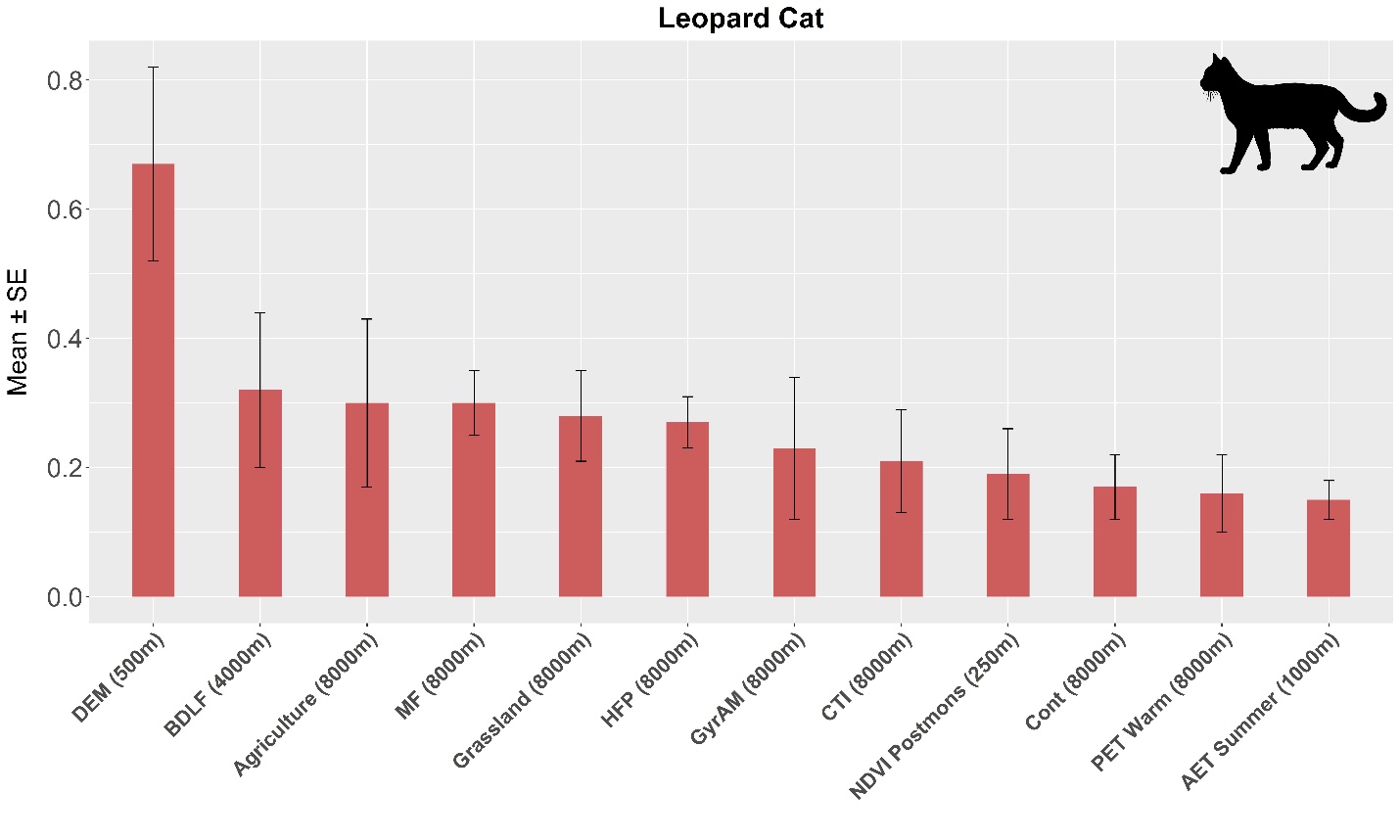

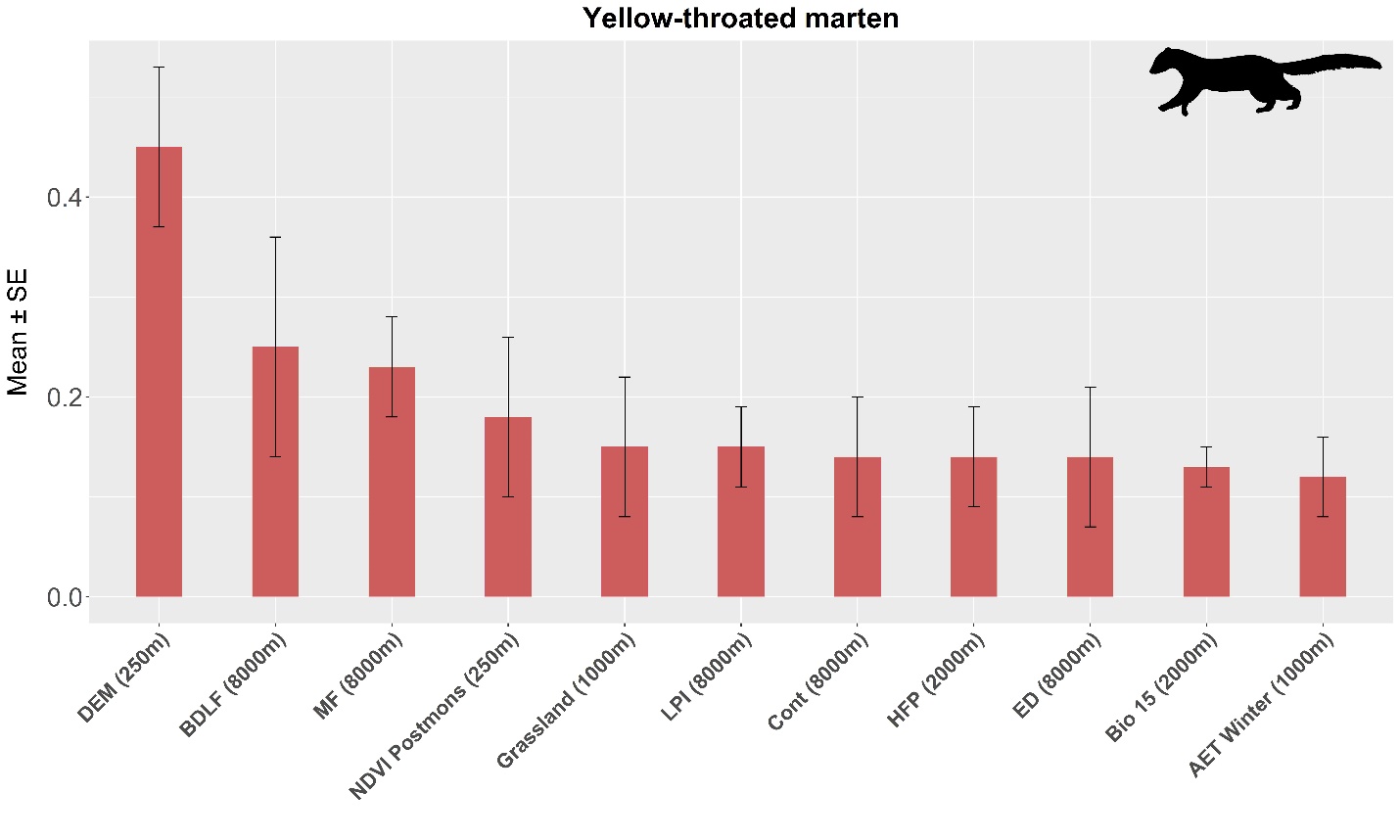

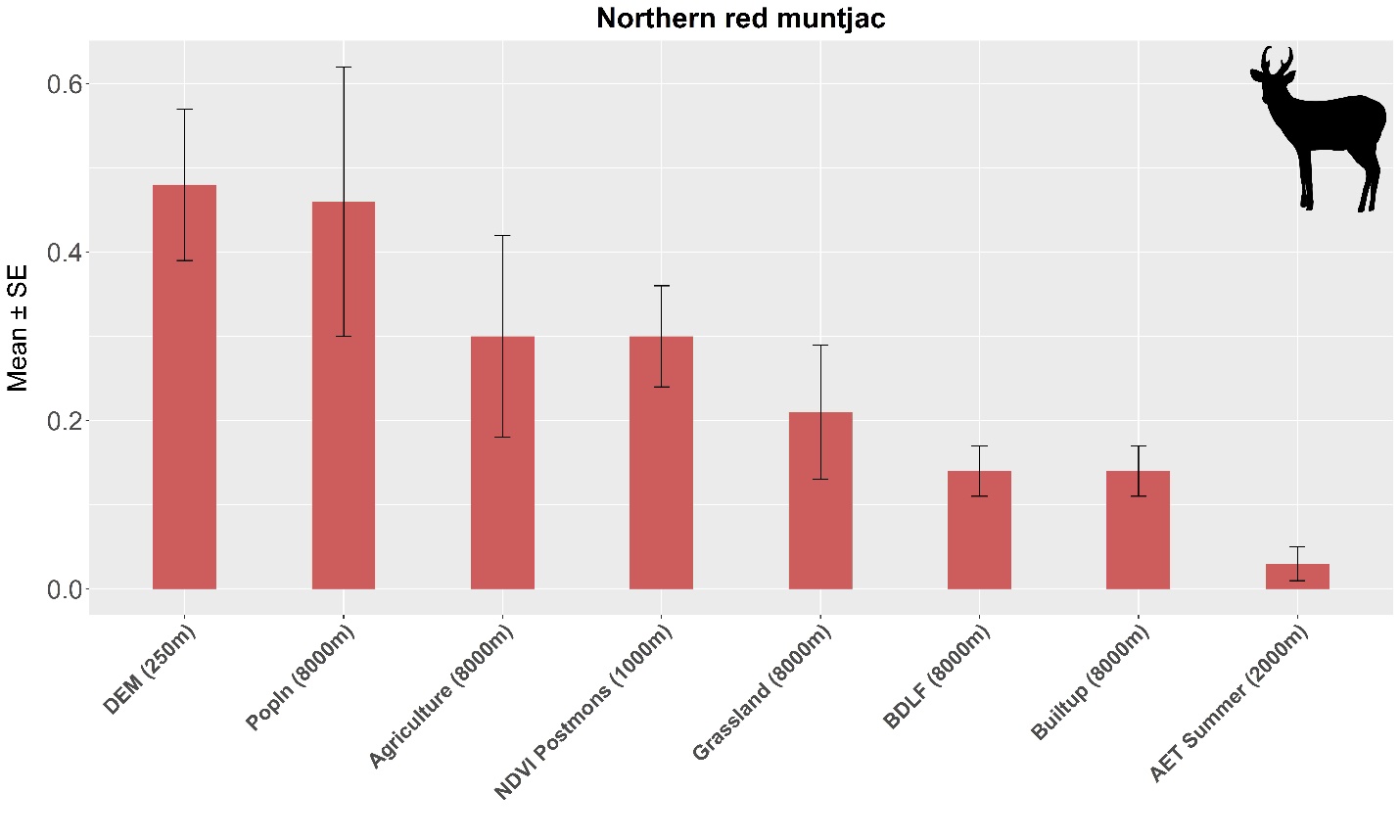

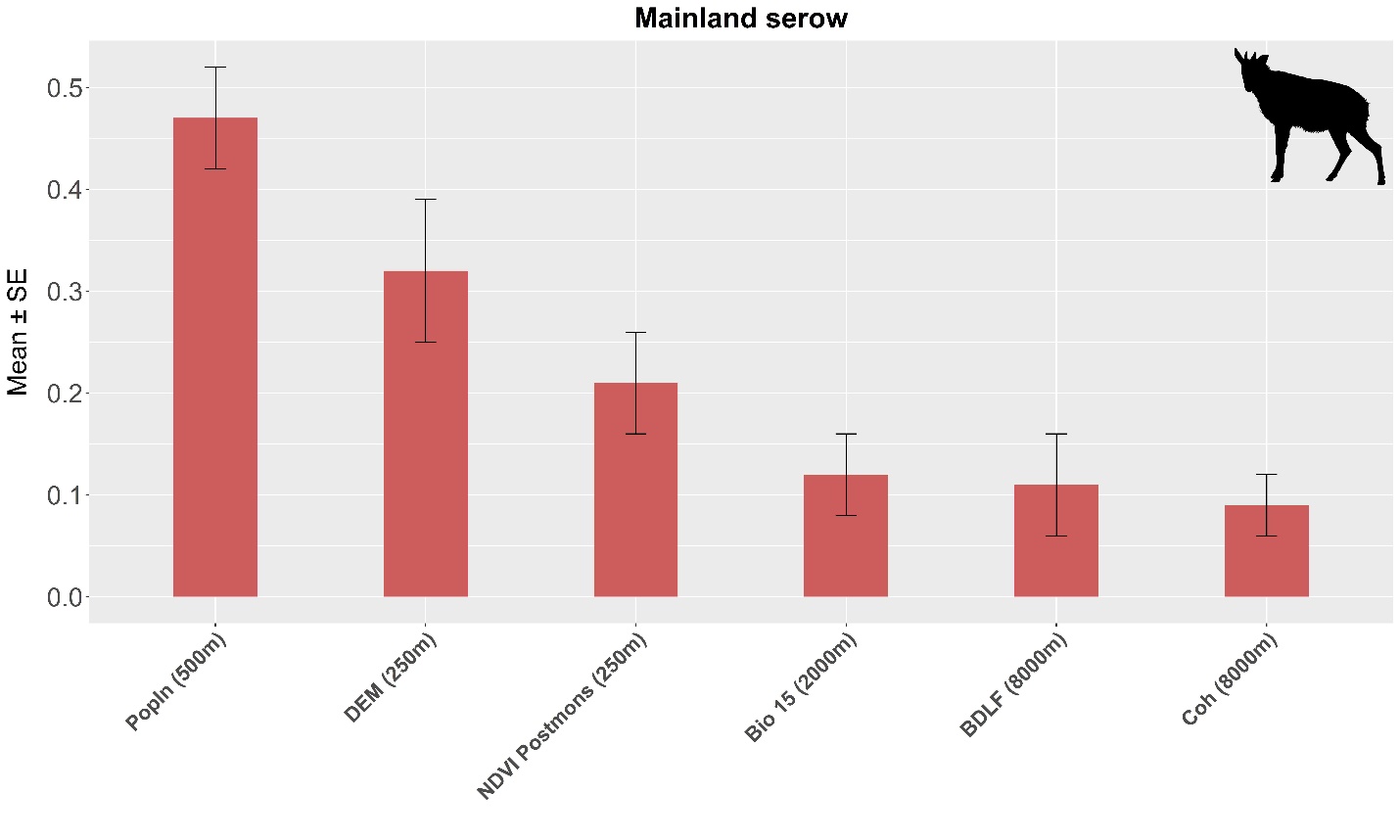

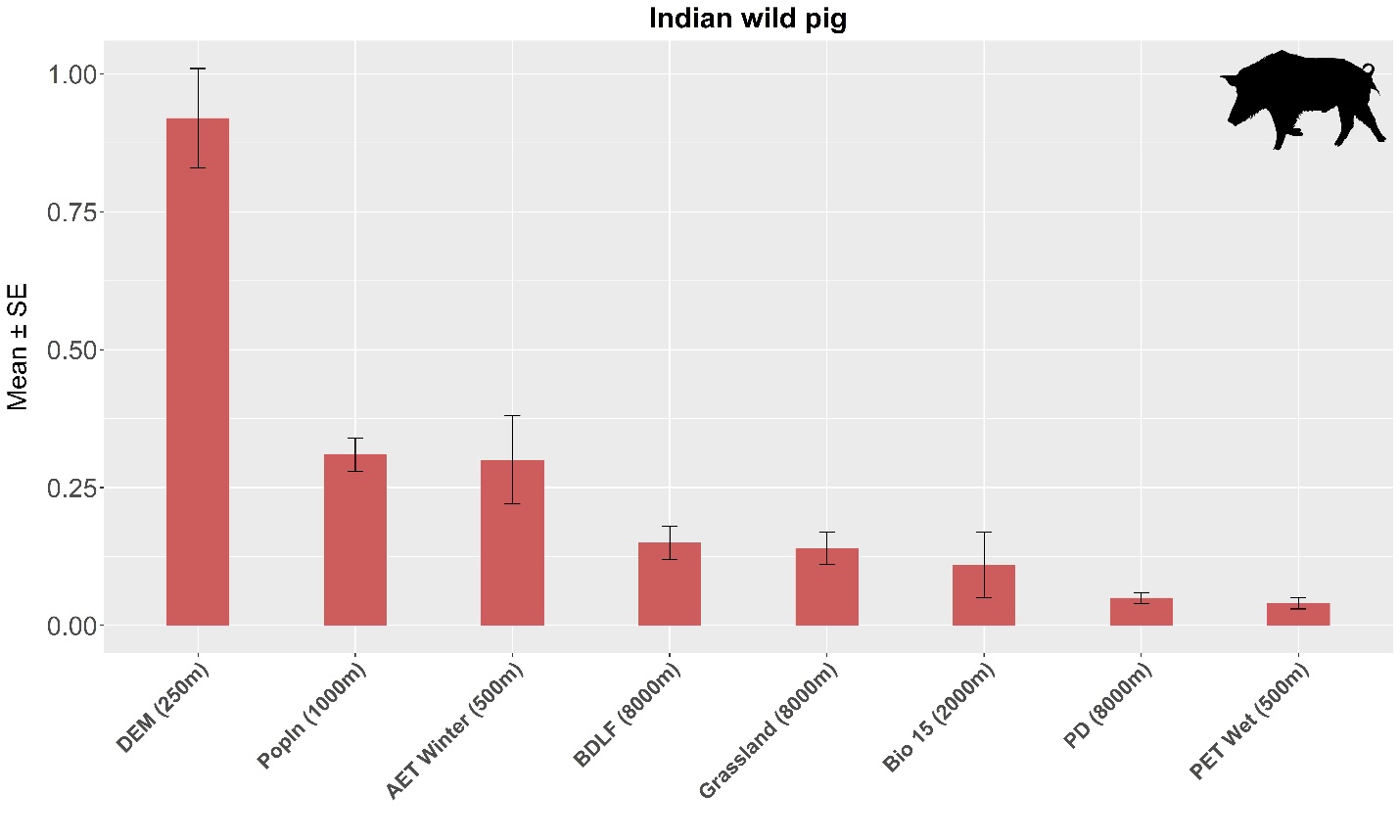

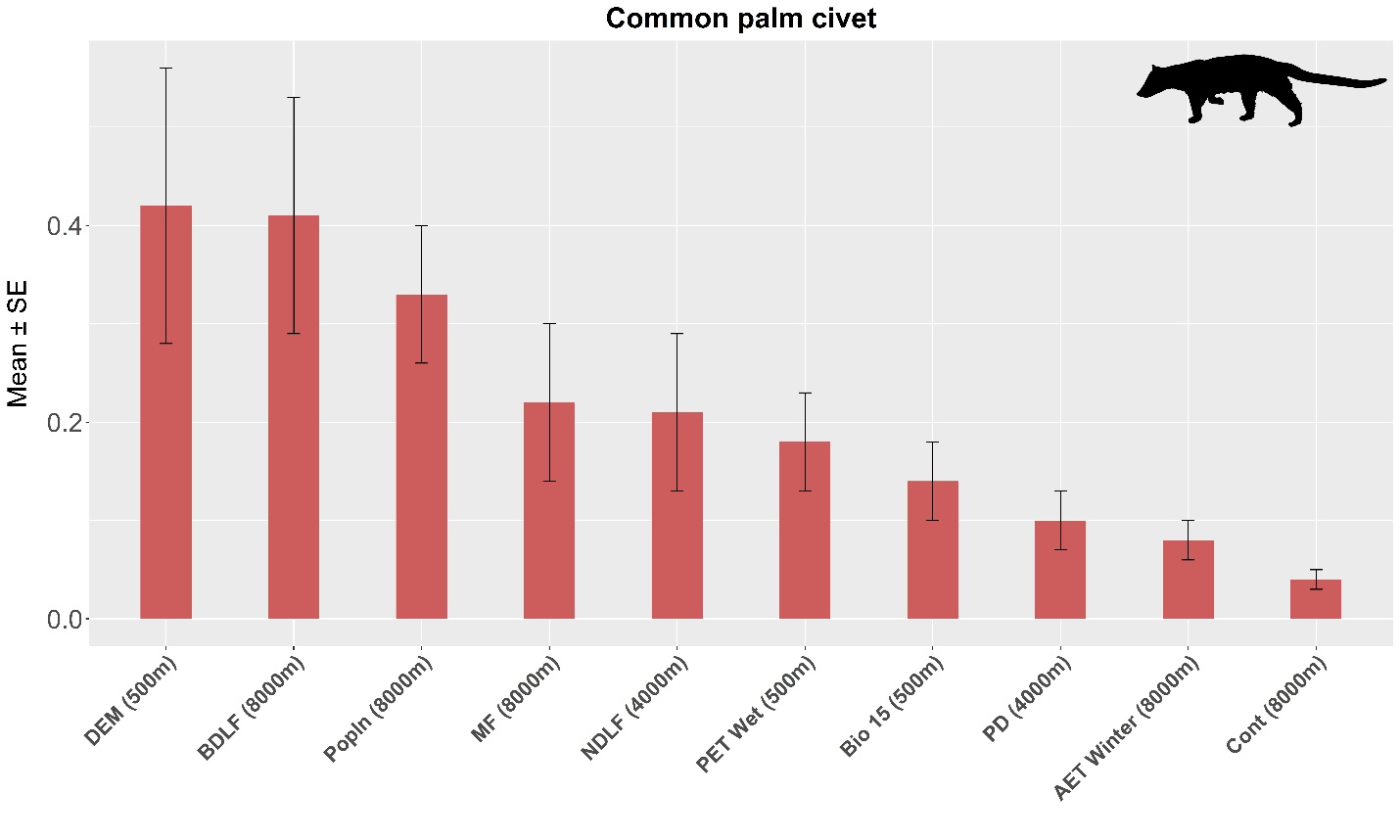

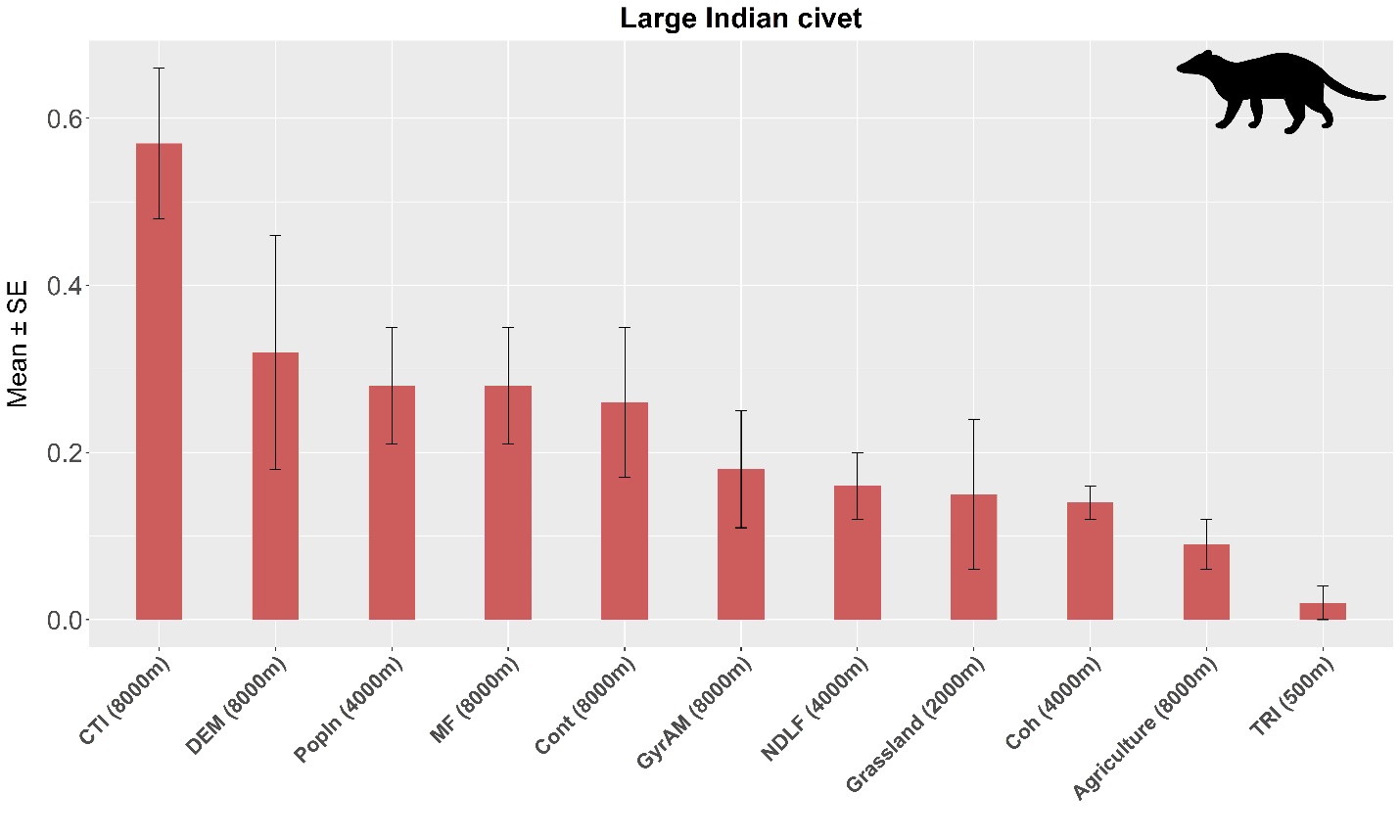

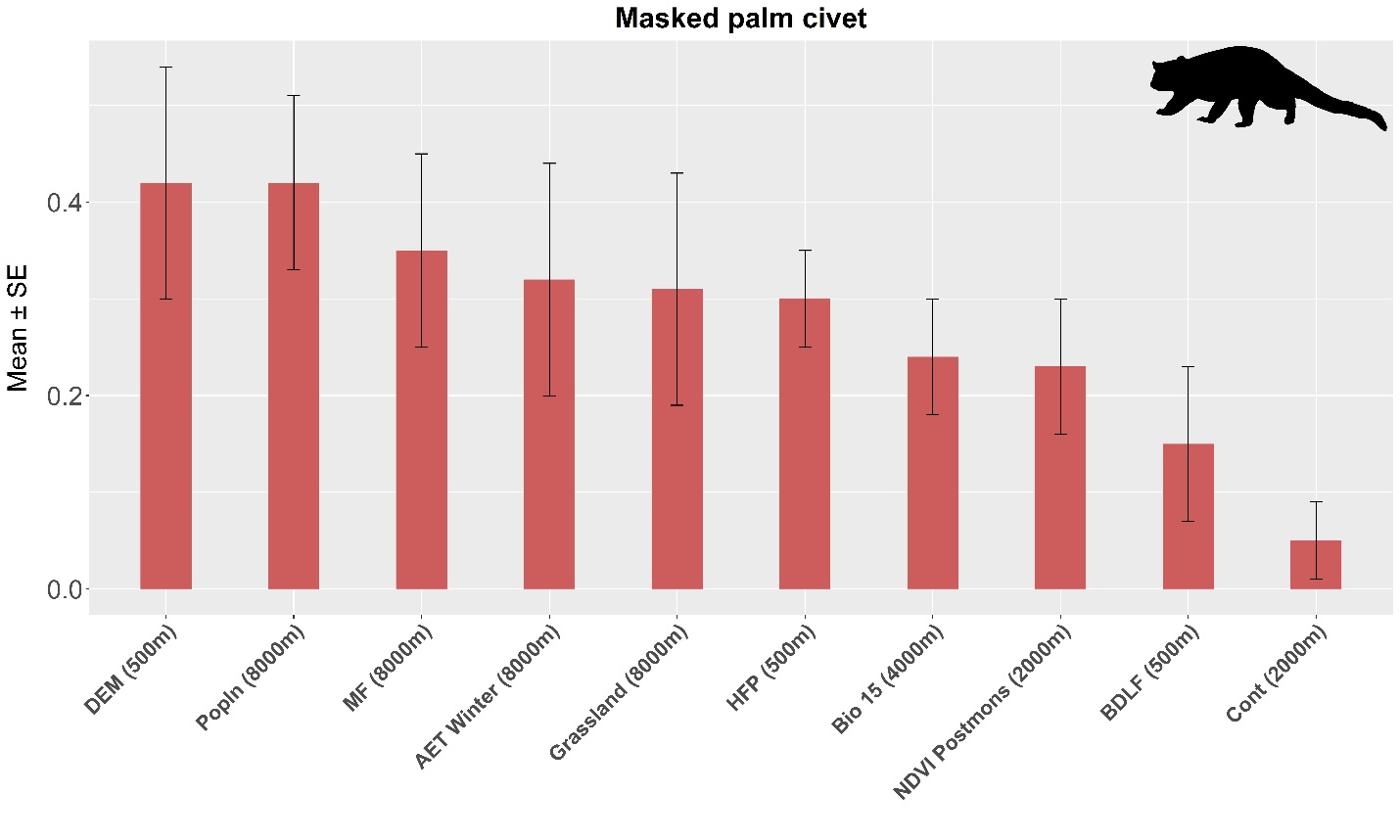

Supplement: Supplementary file 2 — Appendix S2. Variable importance plot for all mammalian species. [file ECE3-15-e71247-s003.docx]
