# Supplementary material for: Understanding Multi‐Scale and Multi‐Species Habitat Selection by Mammals in the Eastern Himalayan Biodiversity Hotspot
Source: Ecol Evol. 2025 Apr 23;15(4):e71247. doi: 10.1002/ece3.71247 (PMC12015752; doi:10.1002/ece3.71247)

**Appendix S3-** **Variable importance response curve**

**Dhole**


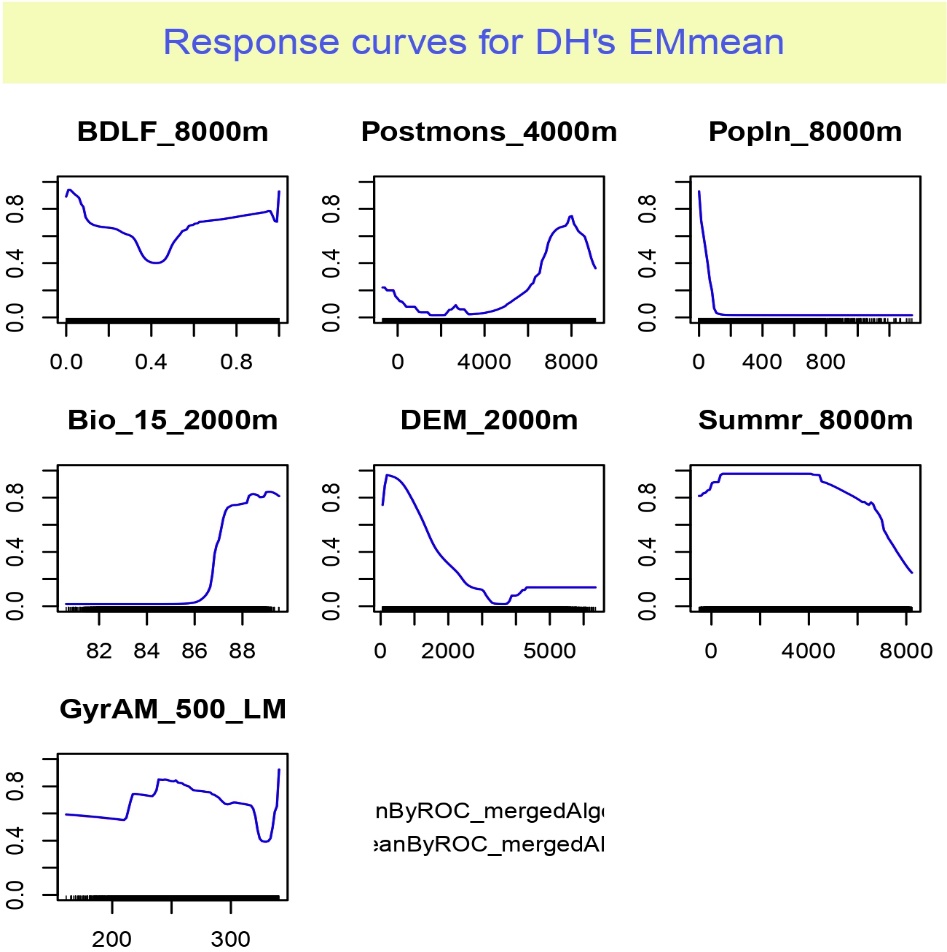


**Asiatic Golden cat**


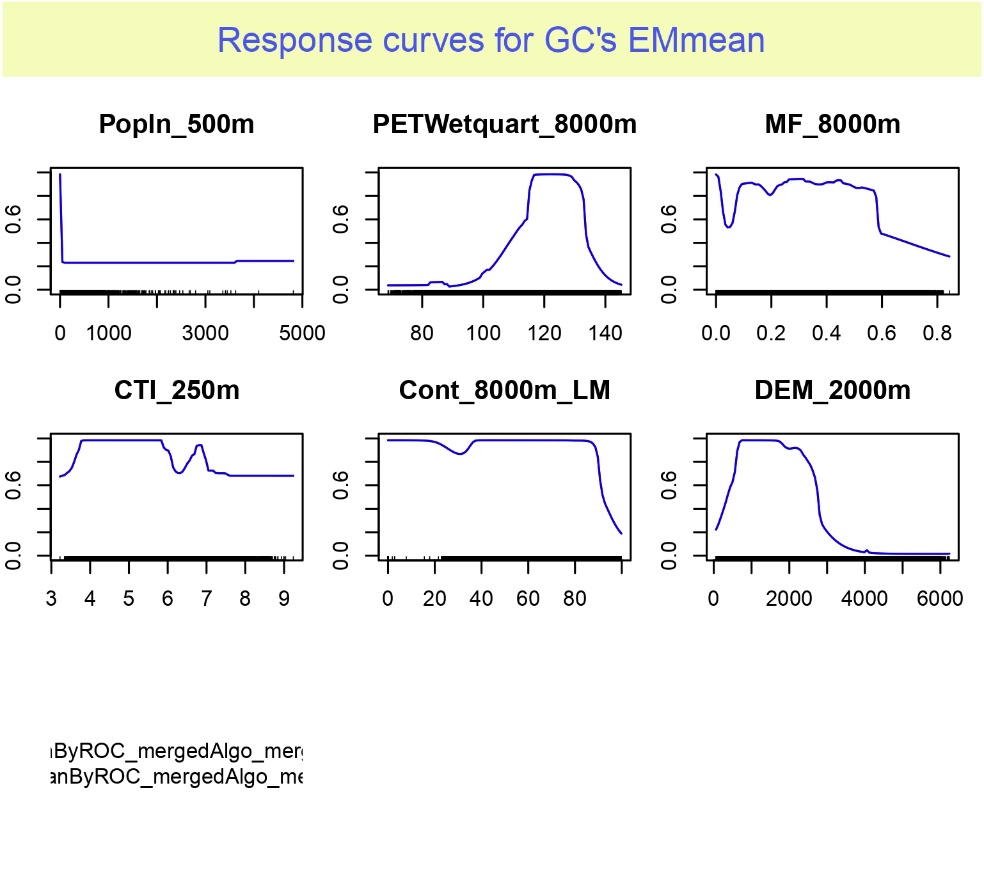


**Leopard Cat**


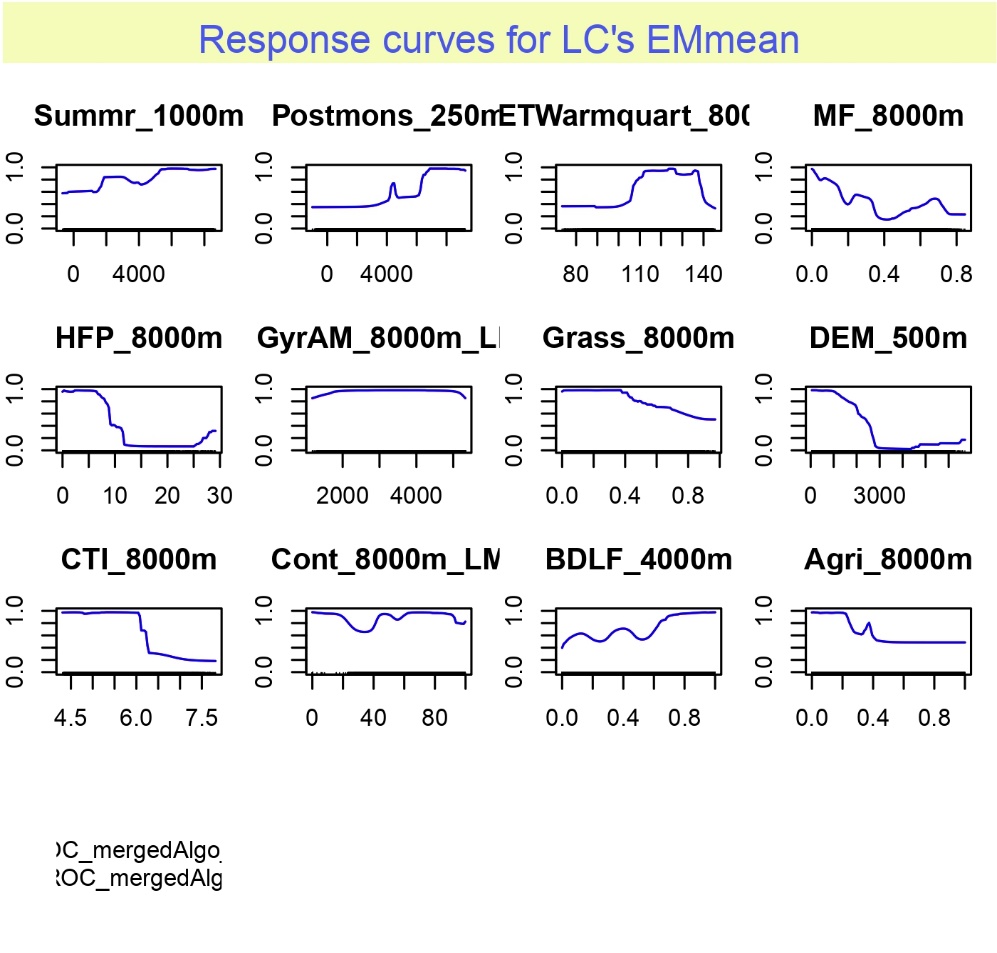


**Yellow-throated Marten**


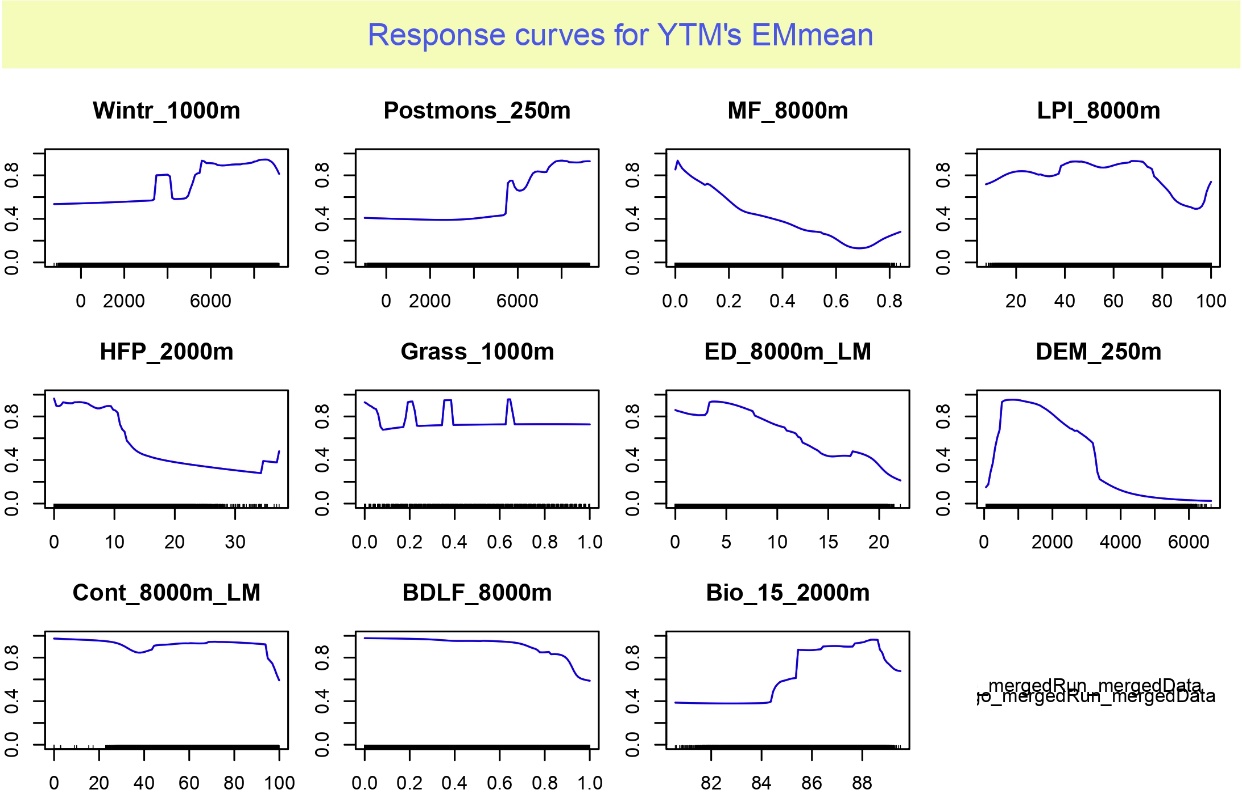


**Northern-red muntjac**


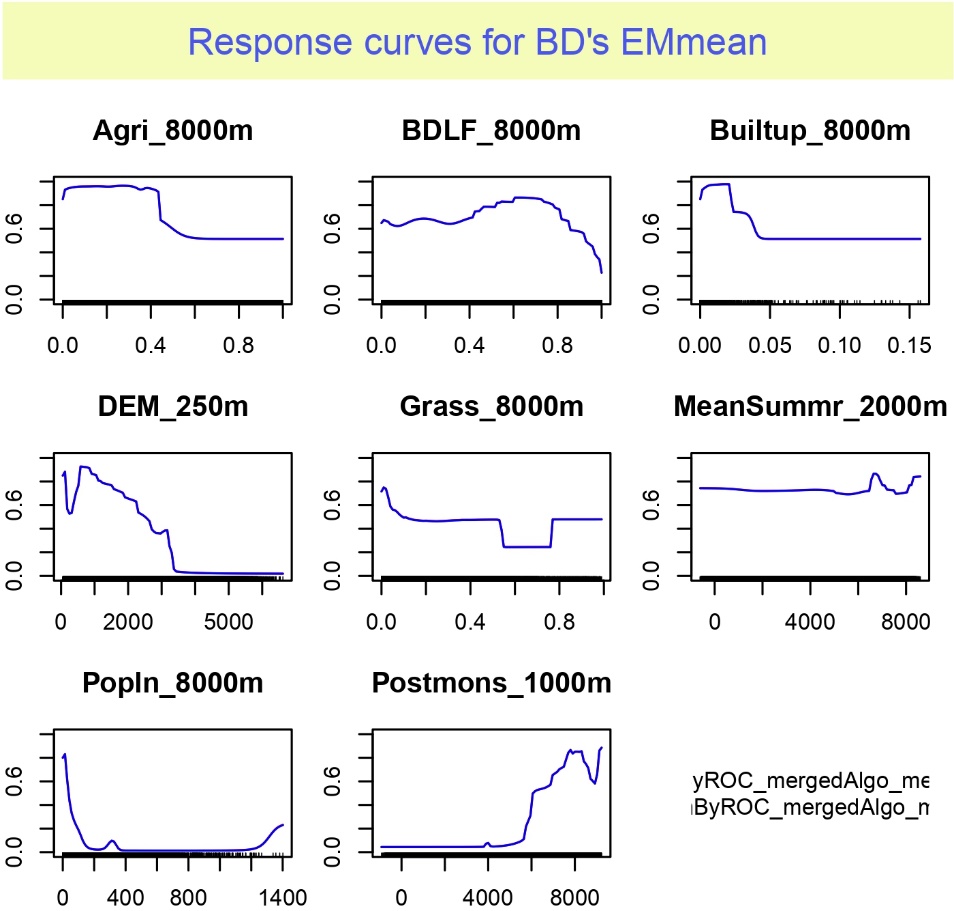


**Mainland Serow**


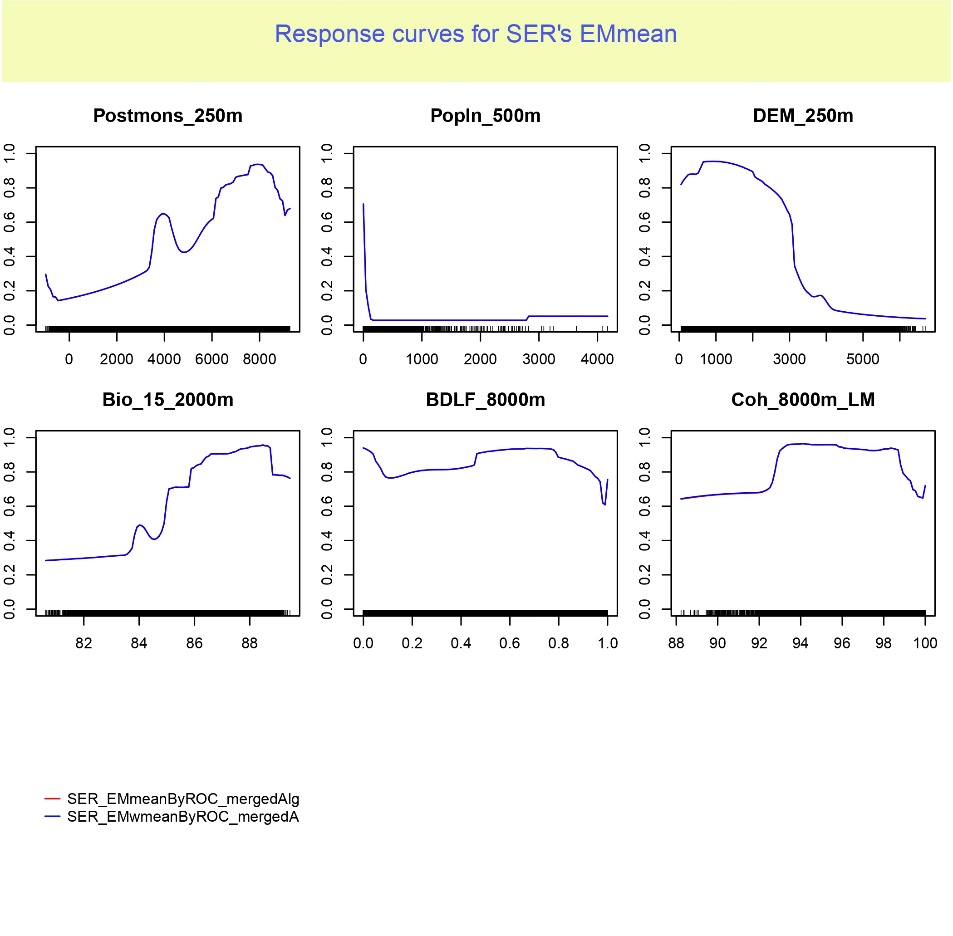


**Indian Wild Pig**


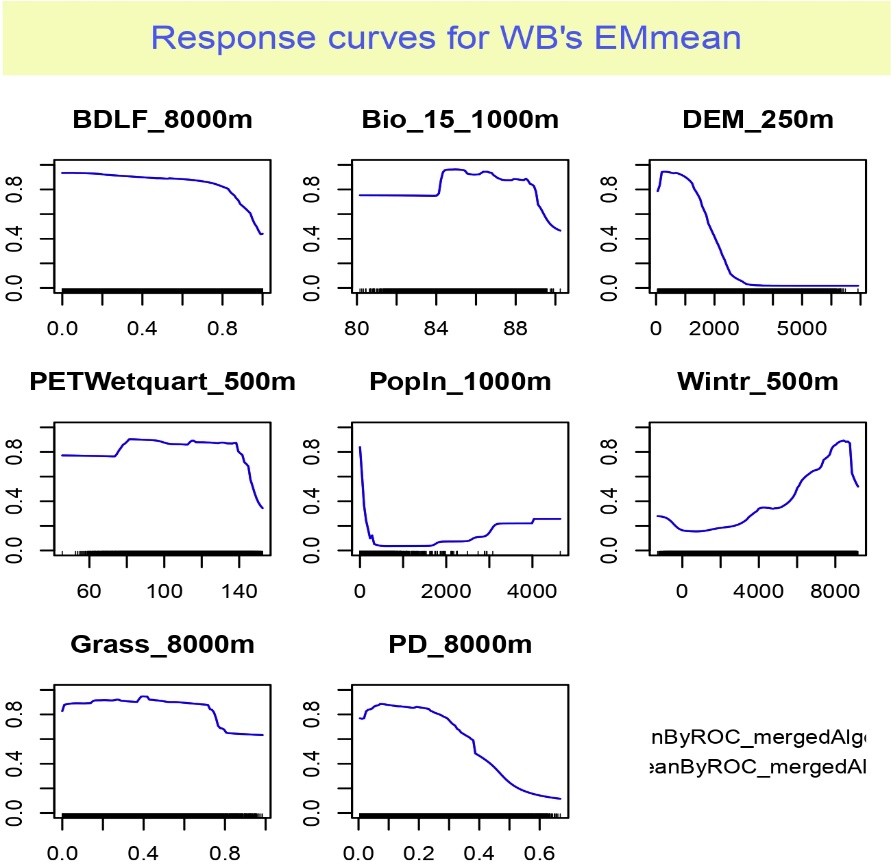


**Common Palm Civet**


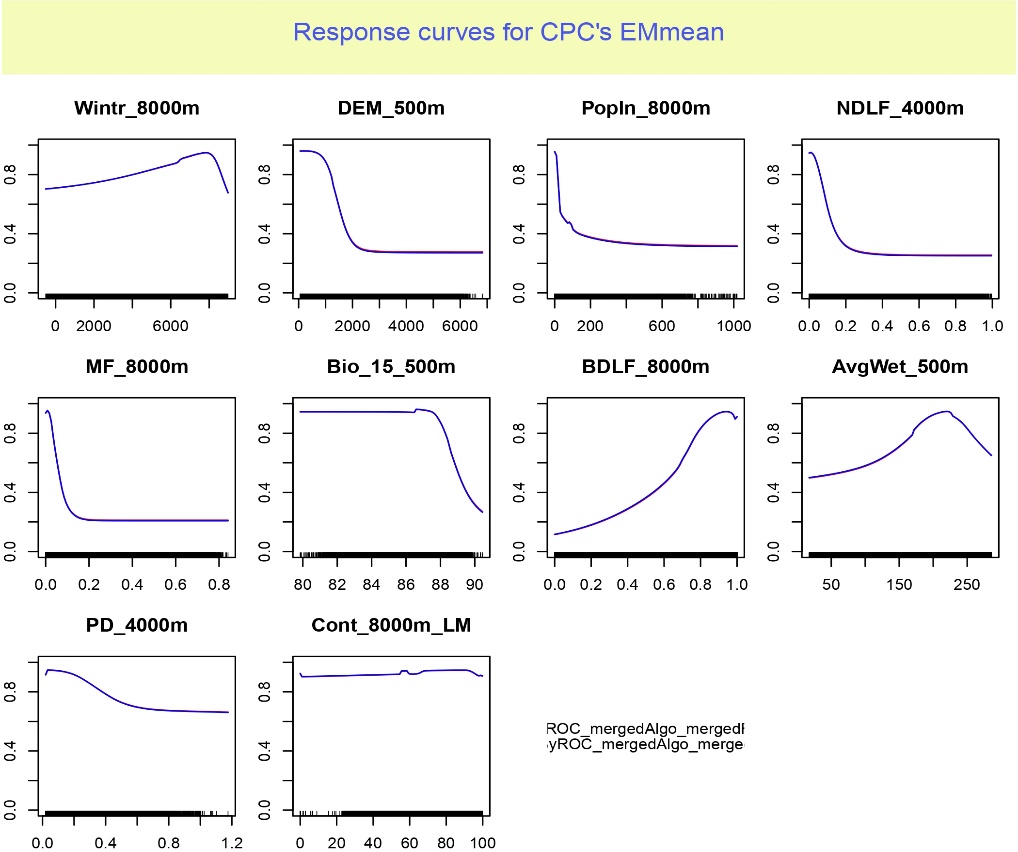


**Large Indian Civet**


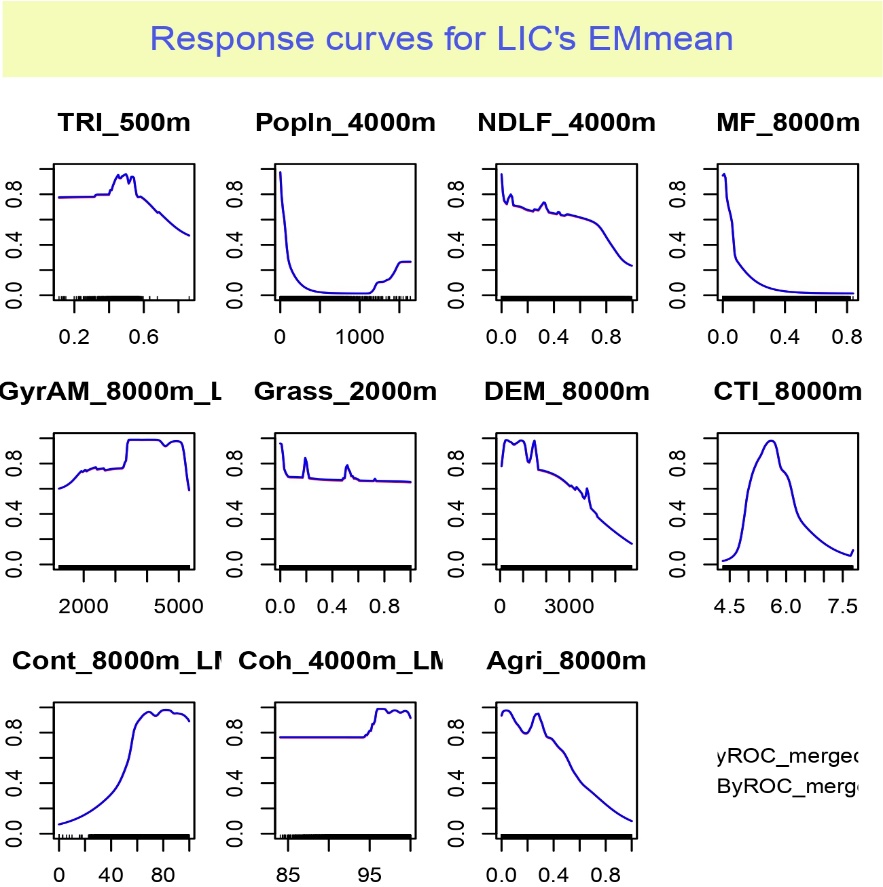


**Masked Palm Civet**


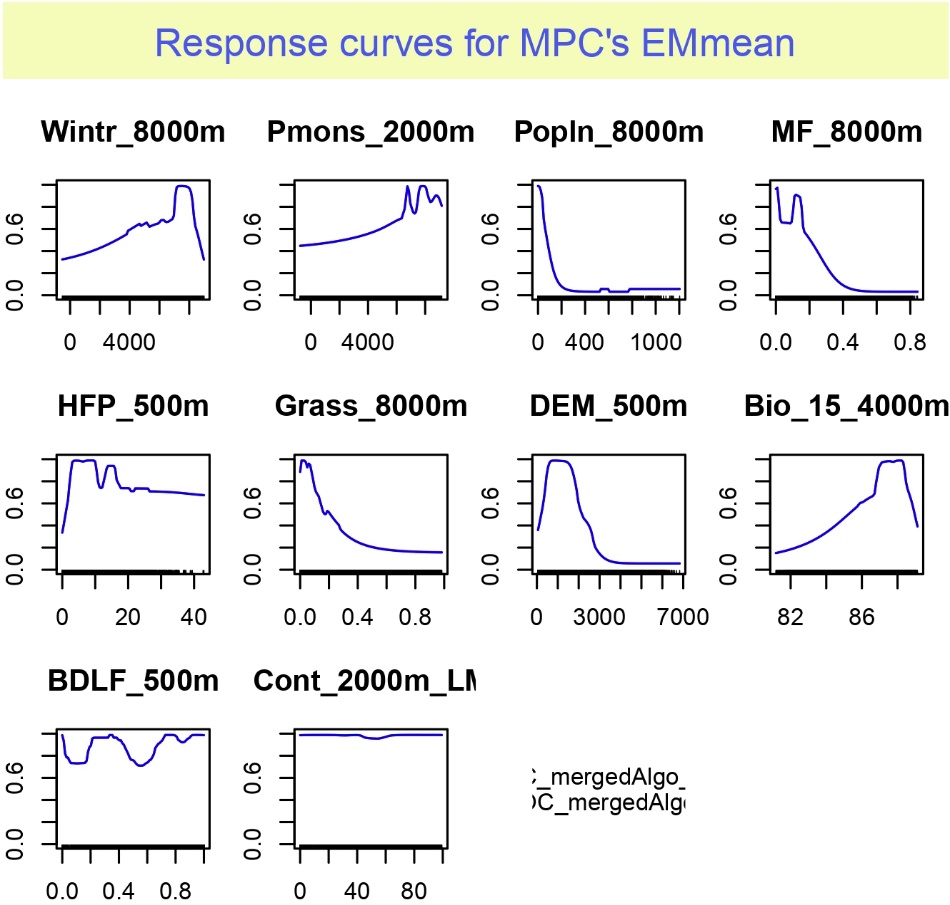

Supplement: Supplementary file 3 — Appendix S3. Variable importance response curve. [file ECE3-15-e71247-s001.docx]
